# Towards green, scalable peptide synthesis: leveraging DEG-crosslinked polystyrene resins to overcome hydrophobicity challenges†

**DOI:** 10.1039/d4ra07484j

**Published:** 2024-12-23

**Authors:** Othman Al Musaimi, Joshua Tomkins, Sarah M. Barry, Alessandra Basso, Xiaokang Kou, Cheng Zhang, Simona Serban

**Affiliations:** a School of Pharmacy, Newcastle University Newcastle upon Tyne NE1 7RU UK othman.almusaimi@newcastle.ac.uk; b Department of Chemical Engineering, Imperial College London London SW7 2AZ UK; c Department of Chemistry, King's College London Britannia House, 7 Trinity Street London SE1 1DB UK; d Life Science Division, Sunresin New Materials Co. Ltd. 710076 Xi'an China simona.serban@sunresin.com

## Abstract

Diethylene glycol dimethacrylate (DEG)-crosslinked polystyrene (PS) resin offers a promising alternative to traditional divinyl benzene (DVB)-PS resin for solid-phase peptide synthesis (SPPS), particularly for challenging sequences with hydrophobic or bulky amino acids. DEG-PS resin's reduced hydrophobicity and enhanced flexibility improve synthesis efficiency, yielding peptides up to 28 residues with higher purities and yields compared to DVB-PS. In various syntheses, DEG-PS outperformed DVB-PS resin, with higher purities and yields for challenging peptides such as ABC analogue (73.2%, 58.3% *vs.* 72.5%, 46.3%) and Thymosin (58.4%, 48.6% *vs.* 54.0%, 39.2%). In addition, DEG-PS resin effectively suppressed common side reactions, such as dipeptide formation, typically encountered with Wang PS-based resins. Incorporating green chemistry principles, DEG-PS enabled the synthesis of complex peptides with satisfactory results using environmentally friendly solvents and reagents. Three challenging peptides; β (34–42), Jung and Redemann (JR), and ABRF 1992 – were synthesized on DEG-PS resin, achieving purities of 41.4%, 41.0%, and 68.0%, and yields of 50.5%, 52.6%, and 56.2%, respectively. These findings highlight DEG-PS resin's advantages for classical, green, and automated SPPS, offering superior performance and scalability for industrial applications.

## Abstract 2

DEG-crosslinked polystyrene (PS) resin is a promising alternative to traditional DVB-PS resin for solid-phase peptide synthesis (SPPS), especially for challenging hydrophobic or bulky sequences.

## Graphical abstract

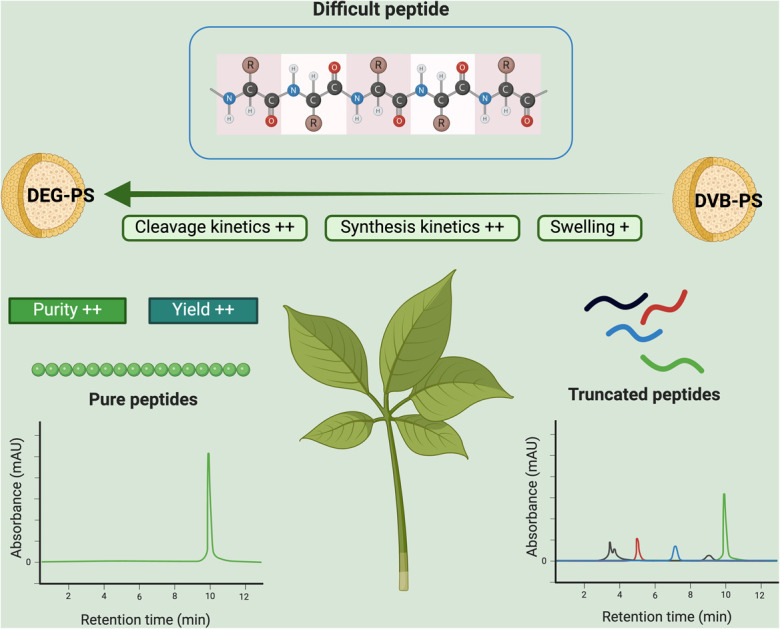

## 1. Introduction

Peptides are becoming a crucial part of the global pharmaceutical industry.^1^ More than 100 peptide therapeutics are currently available, with 31 approved by the United States food and drug administration (FDA) between 2016 and 2023.^2,3^ To produce these new peptide therapeutics with satisfactory yields and purities, pharmaceutical companies must adopt innovative synthetic strategies, including the careful selection of appropriate resins. A variety of resins and materials have been reported for SPPS, but polystyrene (PS) resins are widely accepted as the preferred choice for peptide production.^4,5^ These solid supports are designed to be mechanically stable during the entire synthetic process, swell well in the solvents used for peptide synthesis, are stable at moderate-to-high temperatures, and can be produced in multi-kilogram batches, ensuring the feasibility of different synthetic procedures involved in producing peptide-based active pharmaceutical ingredients (APIs). Furthermore, they have demonstrated excellent performance with green solvents such as 2-MeTHF, *N*-butyl-2-pyrrolidone (NBP), γ-valerolactone (GVL), among others.^6^

Aggregation is a side effect that is often observed during peptide synthesis on solid support. This phenomenon is sequence dependent and is pronounced from the fifth amino acid onwards.^7^ Interchain interactions, such as hydrogen bonding to form secondary structures or hydrophobic interactions resulting in aggregation can reduce the rate of reaction and thus the yield.^8^ The inability of the reaction mixtures, including the amino acid or deprotection solutions, to reach the reactive functional groups on the resin, significantly affects the overall synthetic process. For instance, the incomplete fluorenyl methoxycarbonyl (Fmoc) removal results in truncated sequences that compromise the purity of the target peptide.^8^ Similarly, incomplete coupling reactions lead to lower yield and fuels side reactions. Various approaches have been reported to mitigate this issue, such as disrupting the formation of secondary structures, or by adopting the pseudoproline approach to circumvent the difficulties associated with secondary amino acid acylation.^9,10^

Swelling is crucial to achieve optimal performance with a SPPS resin, as the reaction kinetics in SPPS are diffusion dependent.^11,12^ Resins with greater swelling capacity enhance reaction yield and reduce reaction time by facilitating diffusion.^8,12–16^ Conversely, reduced swelling capacity can trigger undesired peptide aggregation pathways, increasing coupling difficulties.^13,16^ The ability of the resin to swell is highly dependent on its chemical structure and interaction with the reaction solvent. These properties are directly linked to the percentage and type of the crosslinking agent. The incompatibility of apolar PS resin with polar peptide chains assembled on resin and the differing polarity of employed solvents contributes to the difficult synthesis of various peptides.^17^ Therefore, investigating alternative resins for SPPS is crucial.

New cross-linkers have been developed with more flexible polymer backbones, resulting in improved diffusion kinetics. These cross-linkers have been copolymerized with styrene to create PS resins specifically designed for use in SPPS. For example, tetraethyleneglycol diacrylate,^18^ 1,6-hexanediol diacrylate,^19,20^ 1,4-butanediol dimethacrylate,^21,22^ α,ω-bis(4-vinylbenzyl) ethers of mono-, di-, tetra-, or hexa(ethylene glycol),^23^ α,ω-bis(4-vinylbenzyl) ethers of 1,4-butanediol or polytetrahydrofuran,^24^ and others like tri(propylene glycol)glycerolate diacrylate^25^ and glycerol dimethacrylate.^26^ Resins cross-linked by these compounds have shown high synthetic efficiency in SPPS. Other linkers, such as poly(ethylene glycol) (PEG), are used in resins like ChemMatrix.^5^ Furthermore, to improve the hydrophilic nature of divinylbenzene-PS (DVB-PS) resins, poly(ethylene glycol) (PEG) can be grafted onto the resin, as seen in resins like Champion and TentaGel.^5,27^ They are widely used in peptide synthesis due to their improved performance.^28^

Among the alternative cross-linkers previously mentioned, PEG and PEG grafted onto the resin are the most widely considered in peptide synthesis endeavors. However, the main concern with these resins is their excessive swelling. Despite their improved reaction kinetics and ability to synthesize challenging peptides, they pose challenges in industrial settings, as their significant expansion requires large-volume reactors. For instance, ChemMatrix, a polyethylene glycol (PEG)-based resin, although effective in delivering high-purity difficult peptides, is less favored in industrial applications because of these volumetric constraints.^5^ PEG-PS, Champion support, and TentaGel, which are polyethylene glycol-PS graft solid supports, face similar challenges in industrial applications. Although these resins offer improved reaction kinetics and can enable the synthesis of difficult peptides, their significant swelling can require larger volume reactors, making them less practical for large-scale production.^27,29,30^

To the best of our knowledge, the only known example of utilizing DEG-PS resin in peptide synthesis was reported by Wang and coworkers.^31^ They demonstrated superior quality in the synthesis of the ACP peptide on DEG-PS and triethylene glycol dimethacrylate (TEG), compared to synthesis on DVB-PS resin.^31^ Encouraged by the promising results from their study, we felt compelled to further investigate the advantages of this type of resin and explore its potential for use in green SPPS (GSPPS).

**Fig. 1:**
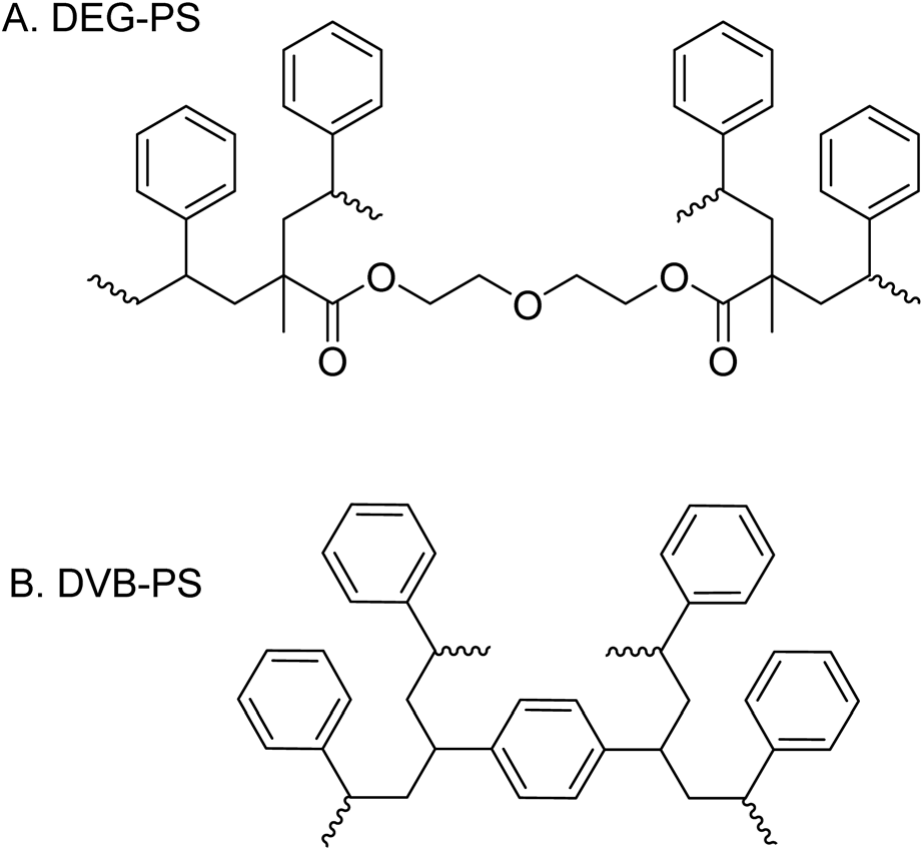
Chemical structures of DEG-PS and DVB-PS resins.

DEG and TEG are cost-effective cross-linkers that enhance the properties of PS resins. Dimethacrylate (DMA) is preferred over diacrylates because polymethacrylates exhibit greater chemical stability than polyacrylates. Moreover, DMAs have favorable copolymerization parameters with PS, leading to more uniform crosslinking, that results in resins with improved structural integrity and performance in SPPS applications (Fig. 1).^31^

Here, we evaluate the application of DEG-PS resin in the synthesis of challenging peptide sequences using conventional, green, and automated methodologies. We focus on peptides that incorporate a variety of demanding amino acid residues, including hydrophobic (*e.g.*, Ala, Ile, Val), sterically hindered (*e.g.*, Arg), and β-branched amino acids (*e.g.*, Ile), which present well-known challenges in the acylation process. The study aims to demonstrate the potential of DEG-PS resins to overcome these challenges, while expanding the scope of synthetic methodologies for peptide production.

## 2. Results and discussion

### 2.1 Resins preparation

According to the procedure reported in the experimental section a series of DEG-PS was manufactured using suspension polymerization. The amount of DEG incorporated was 3% w/w, while DVB was 1% w/w, corresponding to a molar percentage of 1.31% for DEG and 0.8% for DVB. For all resins, SEM analysis revealed that the particle size of the obtained material ranged from 75 to 150 microns.

### 2.2 Scanning electron microscope (SEM)

**Fig. 2:**
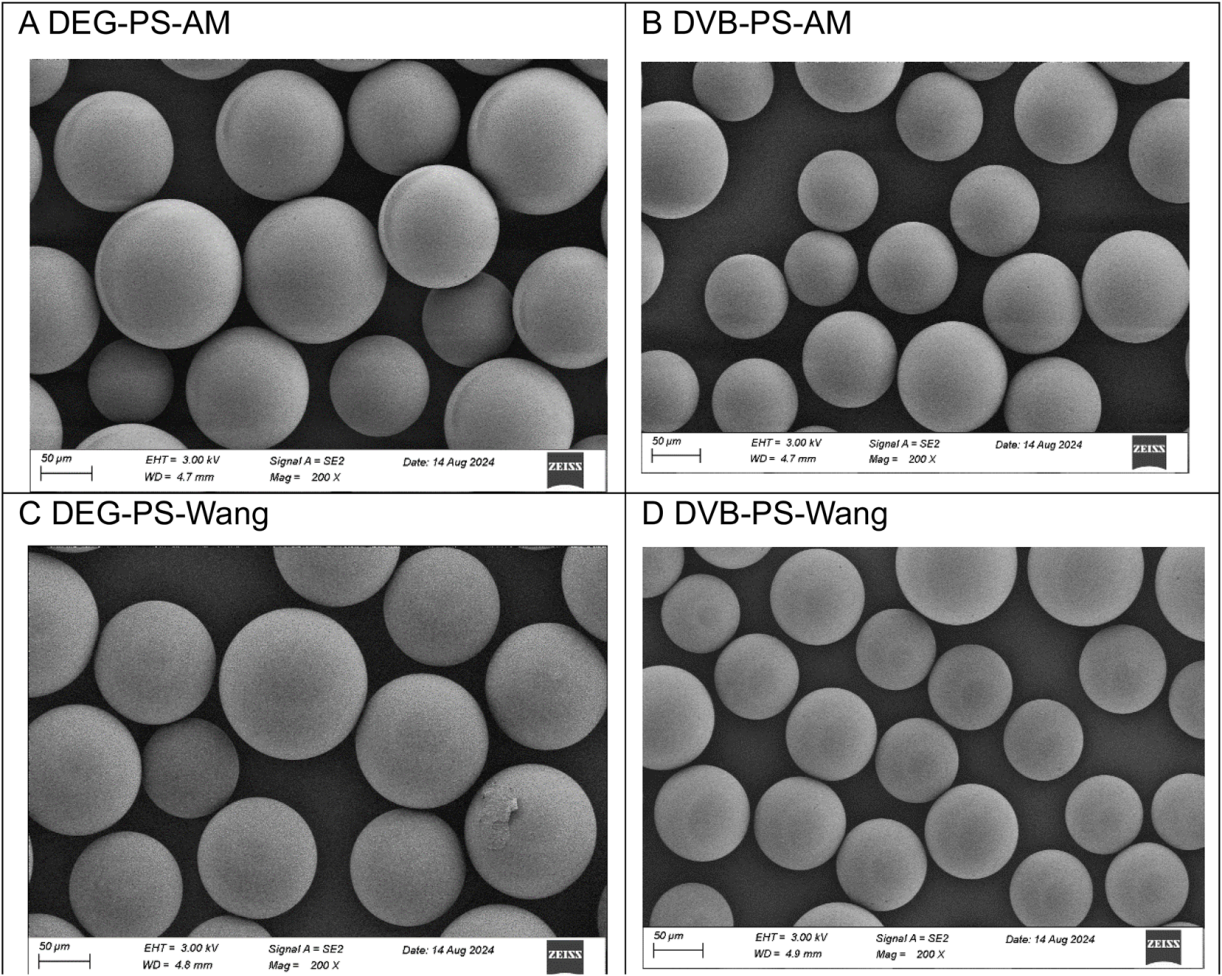
SEM images of DEG- *versus* DVB-PS resins comprising AM and Wang linkers. Scale 50 μm.

1.3% DEG-PS and 1% DVB-PS, AM, and Wang resins were synthesized *via* suspension polymerization and subsequently characterized using SEM. The SEM analysis revealed very similar bead surfaces for all four samples, indicating that, when dry, there are no significant visual differences between the resins. Furthermore, the suspension polymerization process produced uniform particle sizes across all four synthesized resins (Fig. 2).

Based on the previous data, we were motivated to explore the potential of DEG-PS in SPPS and to highlight any advantages it may have over DVB-PS.

### 2.3 Swelling

**Fig. 3:**
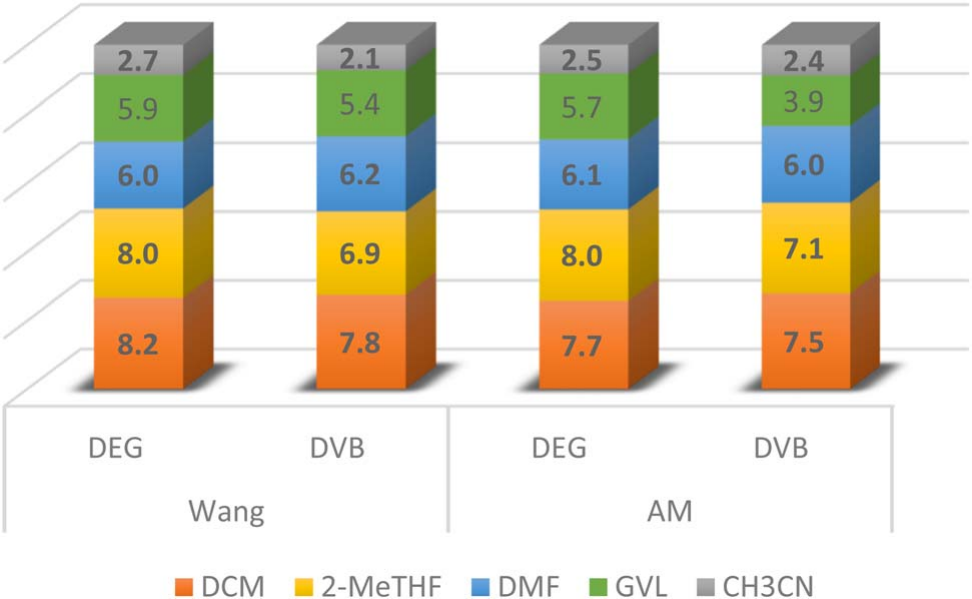
Swelling (g mL^−1^) of DEG-PS *vs.* DVB-PS Wang and AM resins.

We established the swelling of DVB-PS and DEG-PS, including Wang and AM resin in five solvents as follows, *N*,*N*-dimethylformamide (DMF), dichloromethane (DCM), CH_3_CN, GVL, and 2-methyltetrahydrofuran (2-MeTHF) (Fig. 3).

The swelling data indicates that both resins exhibit similar swelling values in all solvents tested, except for the green solvent, 2-MeTHF, where the DEG-PS resins showed a significantly higher swelling capacity compared to DVB-PS. This increased swelling, reaching 1 mL g^−1^ for both Wang and AM DEG-PS resins, is expected to be a game changer, greatly enhancing the efficiency of the peptide synthesis process when using the GSPPS protocol. To corroborate these findings, we carried out a series of simulation tests.

### 2.4 Computational study of DVB-PS and DEG-PS with 2-MeTHF and trifluoroacetic acid (TFA)

**Fig. 4:**
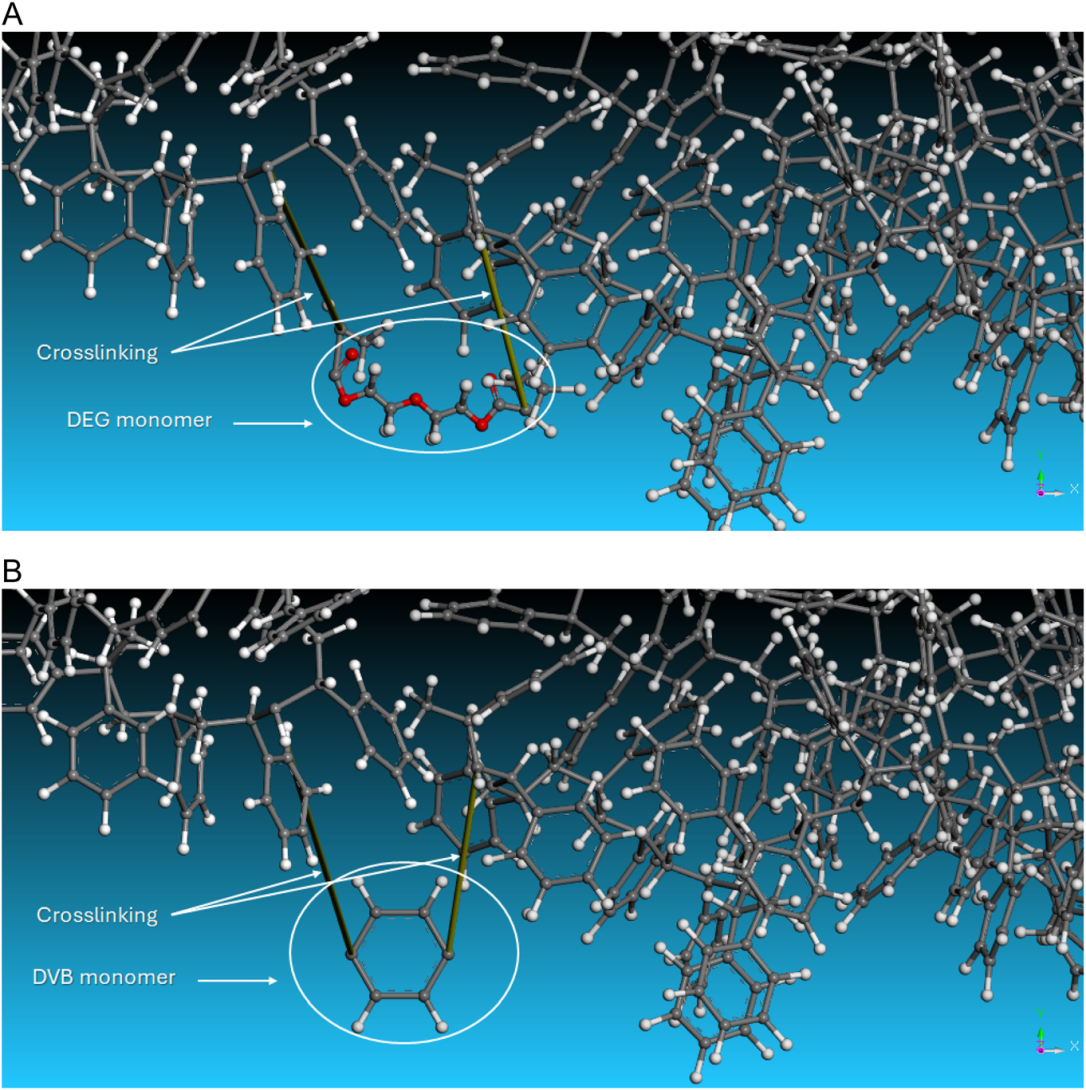
3D Atomistic structures of: (A) 1% DEG-PS, (B) 1% DVB-PS.

Computational screening of molecular interactions is a valuable tool for predicting, validating, and understanding the swelling behavior of SPPS resins.^32^ We aimed to simulate the swelling behavior of the resins in 2-MeTHF, as well as the effects of increasing temperature. We employed the Blends module to determine polymer–solvent interaction parameter (Chi, *χ*) and mixing energy (*E*_mix_). These parameters reflect the swelling capacity between the polymers and solvents; as *χ* and/or *E*_mix_ values increase, the swelling capacity decreases, and *vice versa* (Fig. 4).

Computational analyses of the *χ* and *E*_mix_ values provided valuable insights into the interaction behavior of DEG-PS compared to DVB-PS with 2-MeTHF. In the Flory–Huggins theory, a negative *χ* indicates attraction between the solvent and polymer, a process referred to as exothermic mixing (Table 1).^33^

**Table 1:** *E*_mix_ and *χ* values of 2-MeTHF with DEG-PS *vs.* DVB-PS resins^a^

#	Resin	*E*_mix_ (kcal mol^−1^)		*χ*		Free energy (kcal mol^−1^)	
		25 °C	40 °C	25 °C	40 °C	25 °C	40 °C
1	DVB-PS	−41.67	−53.83	−70.37	−86.54	−11.7	−13.9
2	DEG-PS	−46.45	−87.72	−78.44	−141.03	−13.1	−22.3

a Blends module, base: resin; screen: 2-MeTHF. Geometry optimization done by the Forcite module. “Dreiding forcefield” and “Charge using QEq” were chosen in both tests.

Based on computational simulation results, DVB-PS showed low *E*_mix_ and *χ* values of −41.67 (kcal mol^−1^) and −70.37 (#1, Table 1), respectively, while DEG-PS exhibited even lower values, with *E*_mix_ at −46.45 (kcal mol^−1^) and *χ* at −78.44 (#2, Table 1). To further investigate, we examined the influence of temperature, focusing specifically on 40 °C, which both our screening study and previous work by Albericio suggested as optimal for coupling and Fmoc removal steps when using 2-MeTHF in SPPS.^34^ We observed even lower *E*_mix_ and *χ* values for both resins. However, DEG-PS once again exhibited significantly lower values, with *E*_mix_ at −87.72 (kcal mol^−1^) and *χ* at −141.03 (#2, Table 1), compared to DVB-PS, which showed values of −53.83 (kcal mol^−1^) and −86.54 (#1, Table 1), respectively. Finally, we examined the free energy of both resins at 25 °C and at 40 °C. DEG-PS exhibited lower swelling free energy values compared to DVB-PS, indicating that swelling of DEG-PS is thermodynamically more favorable than DVB-PS. For instance, at both temperatures, DEG-PS exhibited free energy values of −13.1 and −22.3 kcal mol^−1^, while DVB-PS showed values of −11.7 and −13.9 kcal mol^−1^ (Fig. S1 and S2†). By examining the free energy of DEG-PS and DVB-PS, we observed that DEG-PS is more responsive to elevated temperatures, with a significant decrease in free energy of approximately 9.2 kcal mol^−1^, from −13.1 to −22.3 kcal mol^−1^. In contrast, while DVB-PS also responded to the temperature increase, the effect was less pronounced, with a reduction of only about 2.2 kcal mol^−1^, from −11.7 to −13.9 kcal mol^−1^ (Fig. S1 and S2†). We attribute the superior performance of DEG to its flexible linker, in contrast to the rigid cyclic structure of DVB. As a result, the reactive groups attached to the resin are more accessible, leading to improved diffusion of reagents and solvents. In addition, the permanent dipole moment in both DEG and 2-MeTHF can enhance the interactions between them, a property that DVB lacks due to its absence of a dipole.

The improved swelling of the DEG linker also positively impacted the cleavage step, a critical step in SPPS that determines the final outcome of the entire synthetic process. Optimizing this step is essential, as any inefficiency can compromise the entire procedure. To validate this, we performed computational simulations of DEG and DVB linkers with TFA, the primary component in the cleavage mixture. The results showed low and comparable *E*_mix_ and *χ* values for DEG and DVB: −7.73 kcal mol^−1^ and −13.06 for DEG, *versus* −10.39 kcal mol^−1^ and −17.55 for DVB, respectively. This indicates that the DEG–PS interaction is likely optimal, ensuring efficient cleavage reactions.

These findings suggest that DEG-PS exhibits stronger interactions with 2-MeTHF compared to DVB-PS at both room temperature (RT) and elevated temperatures, resulting in improved swelling. Furthermore, this enhanced swelling at 40 °C contributes to better reaction kinetics, thereby increasing the overall efficiency of the synthesis process. In conclusion, with superior acylation kinetics in 2-MeTHF and comparable cleavage kinetics, DEG-PS is expected to outperform DVB-PS resin.

### 2.5 Peptide synthesis

We began with a simple peptide sequence to assess the performance of DVB-PS-Wang *versus* DEG-PS-Wang resins. Leu-Enkephalin, a commonly used reference peptide for evaluating synthetic protocols, was selected for this purpose. Next, we considered synthesizing more challenging peptides including acyl carrier protein (ACP) (65–74), Aib-modified ACP, β (34–42), Jung and Redemann (JR), ABRF 1992, ABC analogue, and Thymosin (ESI Table 1†).

#### 2.5.1 SPPS

As a start, we considered the standard protocol which involves the use of non-green solvents including DMF and DCM for first amino acid incorporation, Fmoc removal, coupling, and washing.

**Fig. 5:**
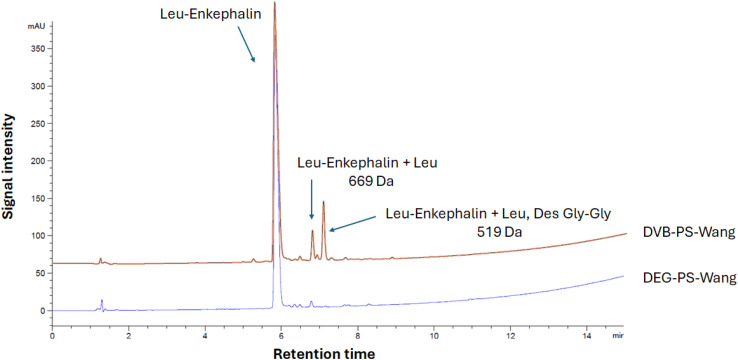
HPLC chromatograms of Leu-Enkephalin synthesized on DVB- and DEG-PS-Wang resins. 5–95% of mobile phase B in 15 min gradient elution monitored at 220 nm. Experimental conditions 1. Leu-Enkephalin, Tyr-Gly-Gly-Phe-Leu-OH.

We synthesized Leu-enkephalin using PS resin crosslinked with DVB and DEG separately. The DEG crosslinker demonstrated better performance compared to DVB. Specifically, the DEG-crosslinked PS resin achieved 96.1% purity for the pentapeptide, whereas the DVB-crosslinked PS resin resulted in 83.6% purity (Fig. 5 and S3–S6†).

When using Wang resin, double incorporation of the first amino acid onto the resin is expected, attributed to the premature removal of Fmoc in presence of catalytic amounts of diamino 4-dimethylaminopyridine (DMAP).^35,36^ While this side reaction was noticeable in the case of DVB-PS Wang resin, it was dramatically suppressed when using DEG-PS Wang resin, from 4.5 to 1.1% (Fig. 5 and S4†). This underscores the improved kinetics of the DEG linker compared to DVB, attributed to its increased flexibility. This flexibility enhances the resin's conformational adaptability, increases surface exposure, and improves swelling behavior, collectively facilitating better solvent accessibility during reactions.

**Fig. 6:**
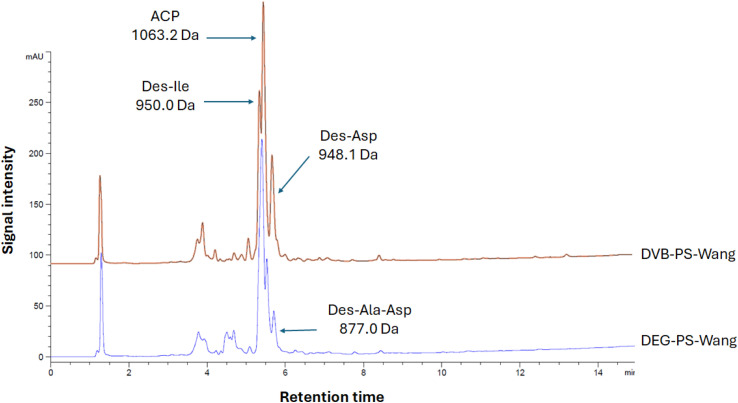
HPLC chromatograms of ACP synthesized on DVB- and DEG-PS Wang resins. 10–60% of mobile phase B in 15 min gradient elution monitored at 220 nm. Experimental conditions 1. ACP, H-Val-Gln-Ala-Ala-Ile-Asp-Tyr-Ile-Asn-Gly-OH.

To get a clearer picture, we carried out the synthesis of ACP (65–74), known as a standard model for challenging new synthetic protocols.^37^ Similarly, we noticed that DEG cross-linked PS resin performed better than the one crosslinked with DVB, where the obtained purities were 39.2% and 68.1%, respectively (Fig. 6). In both syntheses, we observed various side products resulting from lack of incorporation of some amino acids, as confirmed by LCMS data. However, higher purity of the ACP peptide was achieved when using DEG-PS resin (Fig. S7–S14†).

When analyzing the swelling data, we observed that both resins exhibited similar swelling values in DMF. However, DEG-PS showed significantly better swelling in 2-MeTHF compared to DVB-PS (Fig. 3). Because of this difference, we decided to explore the potential added value of DEG-PS's superior swelling in the subsequent GSPPS.

#### 2.5.2 GSPPS

We were motivated to consider 2-MeTHF in our comparison study for two reasons. Firstly, the higher swelling of DEG-PS would boost the overall synthesis efficiency. Secondly, 2-MeTHF is a green solvent,^38–41^ and has proven its suitability as a universal solvent for all steps of SPPS including, loading of the first amino acid, Fmoc deprotection, coupling, washing, and precipitation.^34,42^ While GVL is also a green solvent, it did not result in improved swelling of DEG-PS resin and thus was not considered further. For example, with Wang resin, both DVB- and DEG-PS resins exhibited similar swelling behavior in GVL. However, in the case of AM, DEG-PS showed greater swelling, though not as much as in 2-MeTHF. Notably, we performed computational screening to assess the swelling behavior of DEG-PS in *N*-Butyl-2-pyrrolidone (NBP) as a potential green solvent. While the results were promising, they were inferior to those obtained with 2-MeTHF, as indicated by higher *E*_mix_ and *χ* values: −19.32 kcal mol^−1^ and −32.62 at 25 °C, and −40.76 kcal mol^−1^ and −65.54 at 40 °C, respectively.

**Fig. 7:**
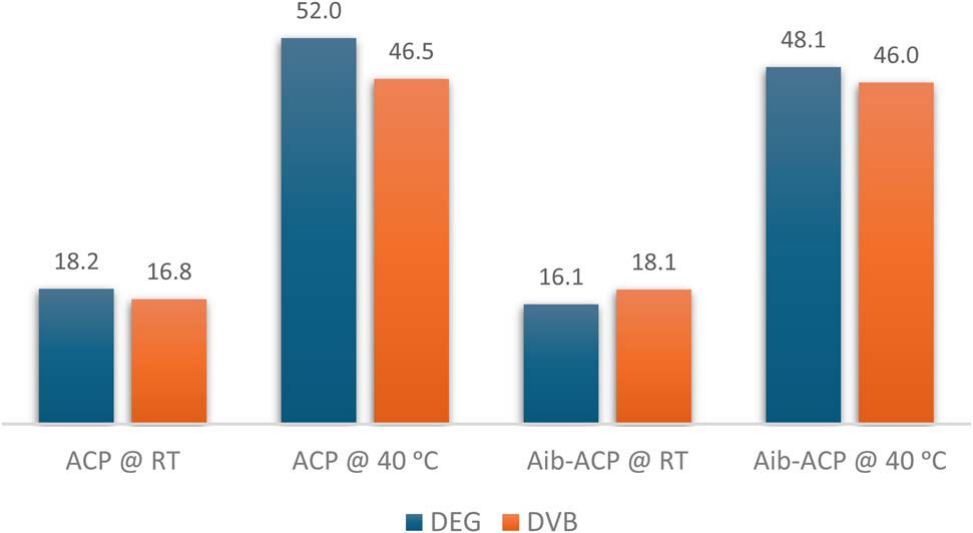
HPLC % purity of ACP and Aib-ACP peptides in 2-MeTHF at RT and 40 °C. HPLC chromatograms were acquired at 220 nm.

We resynthesized ACP and Aib-modified ACP using 2-MeTHF as solvent for SPPS. Both our computational screening data and an earlier study conducted by the Albericio group, found that using a slightly higher temperature (40 °C) with 2-MeTHF results in improved reaction kinetics compared to RT.^34^ Thus, we synthesized the ACP and Aib-modified ACP at both RT and 40 °C to comprehensively explore the behavior of DEG-PS under these conditions. This would allow us to evaluate its performance across a broader temperature range (Fig. 7 and S15–S24†).

From the data shown in Fig. 7, the superiority of DEG-PS resin is evident. Even at RT, DEG-PS resin outperformed DVB-PS, producing ACP peptide with purities of 18.2% and 16.8%, respectively. Higher temperatures are crucial for improving reaction kinetics. This was observed in both resins studied; however, the DEG polymer appears to be more responsive to elevated temperatures compared to DVB, resulting in better overall reaction kinetics. Conducting the reaction at a slightly higher temperature, 40 °C, significantly enhanced the quality of both peptides synthesized on either resin. However, DEG-PS resin was superior to DVB-PS. Ultimately, the best purities of both peptides were achieved when synthesized on DEG-PS resin, at 40 °C, with ACP at 52.0% and Aib-ACP at 48.1%.

The polymer's responsiveness to temperature changes is essential for improving reaction kinetics. DEG-PS has shown this capability, a finding further supported by our computational data.

#### 2.5.3 Difficult peptide sequences

**Fig. 8:**
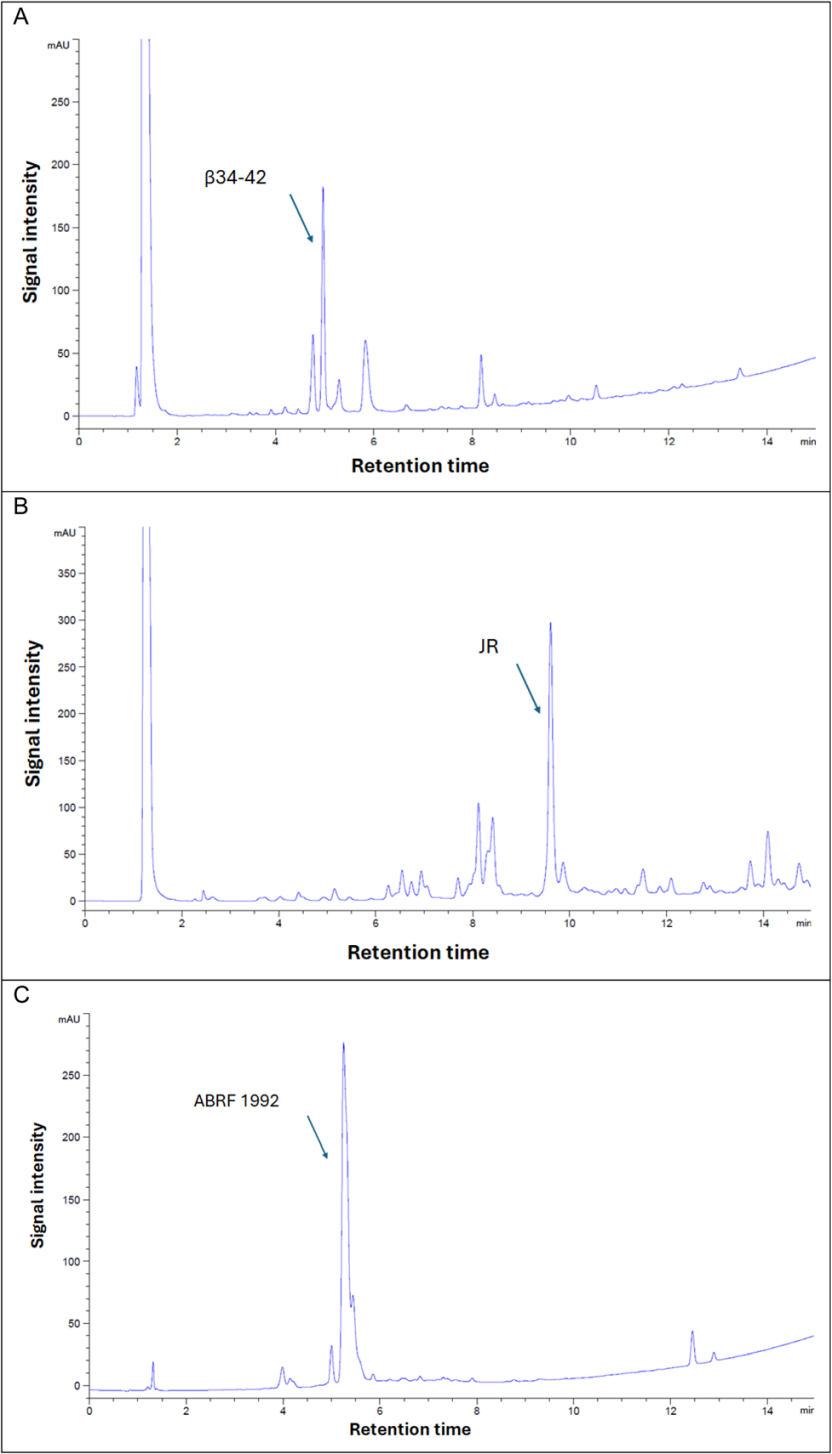
HPLC analysis at 220 nm of β (34–42), purity achieved 41.4%; JR, purity achieved 41.0%; both synthesized on DEG-PS Wang resin, and ABRF 1992, purity achieved 68.0% synthesized on DEG-PS rink-amide-AM resin and analyzed directly after cleavage/deprotection. 5–95% of mobile phase B in 15 min gradient elution. Experimental conditions 1. β (34–42), H-Leu-Met-Val-Gly-Gly-Val-Val-Ile-Ala-OH; JR, H-Trp-Phe-Thr-Thr-Leu-Ile-Ser-Thr-Ile-Met-OH; ABRF 1992, H-Gly-Val-Arg-Gly-Asp-Lys-Gly-Asn-Pro-Gly-Trp-Pro-Gly-Ala-Pro-Tyr-NH_2_.

**Fig. 9:**
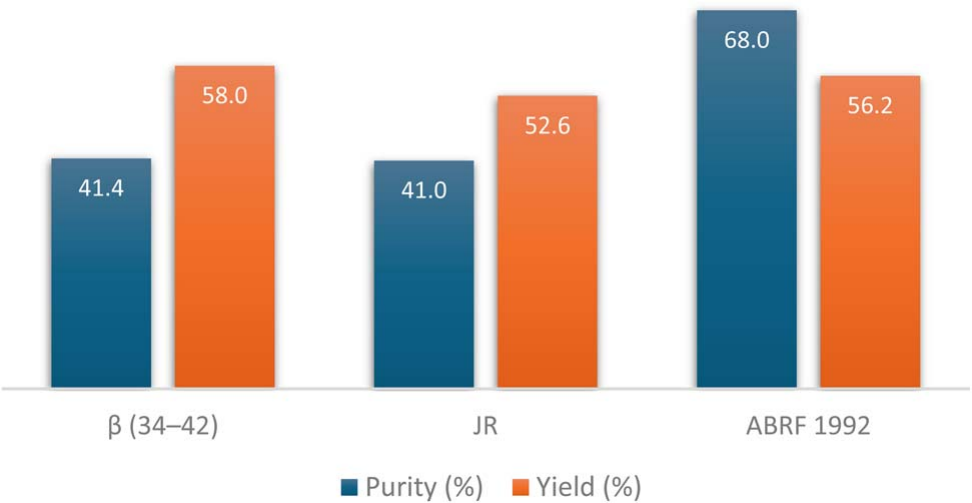
HPLC % purity and isolated yield of β (34–42), JR, and ABRF 1992 peptides synthesized on DEG-PS resin. HPLC chromatograms were acquired at 220 nm.

To validate the effectiveness of the DEG-PS resin, three additional challenging peptides were manually synthesized using the established green conditions. These include: (i) β (34–42), an extremely insoluble, hydrophobic peptide with an antiparallel β-sheet structure, representing the C-terminal nonapeptide fragment of the β-amyloid protein β (1–42), (ii) JR, the C-terminal decapeptide fragment of a 26-mer peptide known for its challenging synthesis, and (iii) ABRF 1992, a peptide identified as difficult by the Association of Biomolecular Resource Facilities (ABRF) (Fig. 8 and S25–S33†).^43^ These three peptides were synthesized under green conditions, demonstrating satisfactory purities and the adaptability of green SPPS methodologies. In each case the target peptide was successfully synthesized with very good purity (Fig. 9). This achievement is notable given that these peptides are known to be extremely difficult to obtain using DVB-PS resin, unless an automatic synthesizer with extended coupling and deprotection steps is employed.^44^

Evidently, β (34–42) and JR were the most challenging peptides, as both resulted in the lowest purities during synthesis. β (34–42) peptide proved to be particularly challenging, which can be attributed to its highly hydrophobic sequence. Furthermore, this peptide was found in the ether layer, which is commonly used to precipitate peptides following the global cleavage step. Indeed, the high hydrophobicity also impeded the Fmoc removal step, where 24.7% of the Fmoc-β (34–42) peptide was detected, as confirmed by LCMS data (Fig. S26†). The difficulty in synthesizing JR is evident from the final purity, where multiple deletion sequences were observed, including des-Trp, des-Phe, des-Thr, and des-Trp-Thr, and this as expected with this particular peptide, (Fig. S28–S31†). Similarly to β (34–42), 4.6% of Fmoc-JR peptide was also detected (Fig. S32†).

Fmoc removal is a demanding step in SPPS,^45^ with the challenge of achieving complete removal becoming more pronounced as the peptide chain grows, especially when hydrophobic residues are involved.^13,16^ Intermolecular interactions among the elongating peptide chains create a barrier, obstructing the base from fully interacting with the Fmoc group and leading to partial removal. Over successive cycles, residual Fmoc groups from earlier steps may eventually be removed, but this often results in the formation of truncated peptides, reducing the overall purity and yield. Reducing hydrophobicity is crucial to overcome these challenges, ensuring efficient Fmoc removal, complete reaction progression, and minimized impurities. The data presented in Fig. 9 highlights the effectiveness of DEG-PS resin in addressing these obstacles, demonstrating its utility for synthesizing challenging peptides with the GSPPS protocol.

#### 2.5.4 Automatic synthesis using DMF

**Fig. 10:**
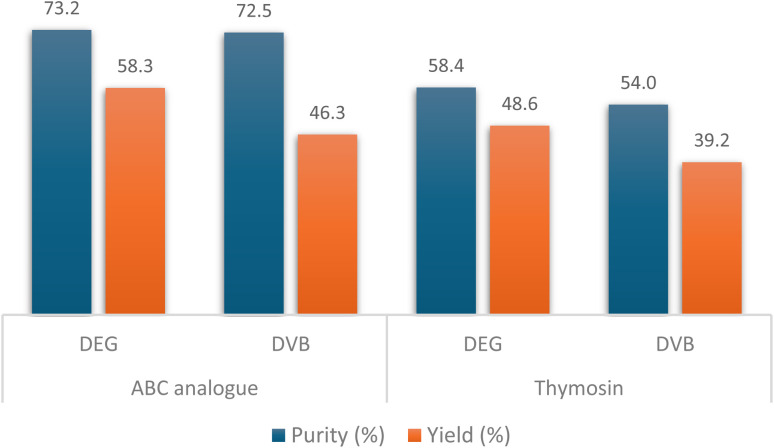
HPLC % purity and isolated yield of ABC analogue and thymosin peptides on DEG-PS and DVB-PS resins, using automatic synthesizer at 90 °C. HPLC chromatograms were acquired at 214 nm.

To further demonstrate the applicability of DEG-PS and its viability in automatic synthesis, two challenging peptide sequences were synthesized using a microwave-assisted automatic peptide synthesizer: An ABC analogue, a 20-mer peptide that includes the 20 proteinogenic amino acids, and Thymosin, a 28-mer peptide. The same synthetic conditions were considered to synthesize both peptides on DVB-PS and DEG-PS (Fig. 10).

**Fig. 11:**
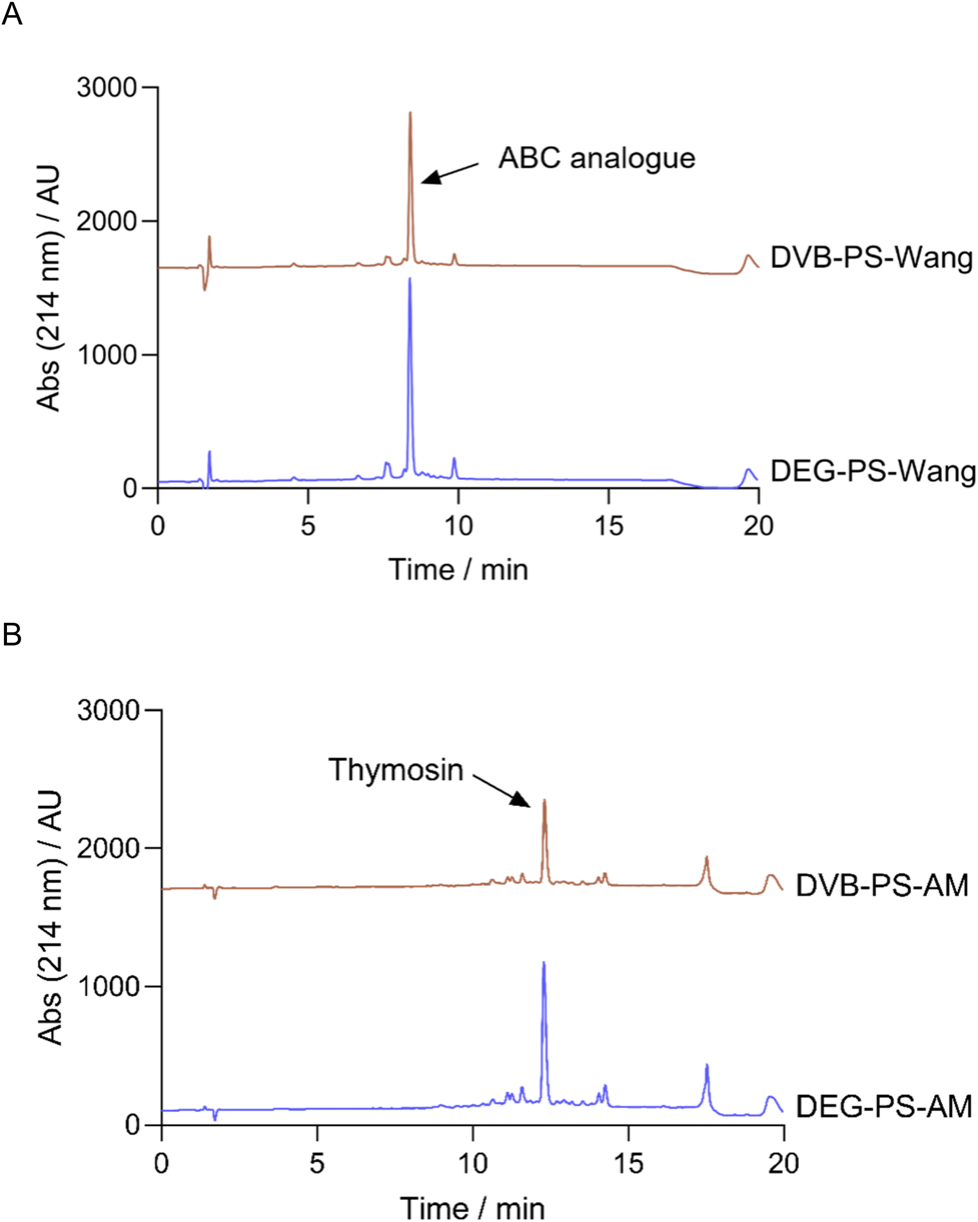
HPLC chromatograms of ABC analogue, automatically synthesized on DEG/DVB-PS Wang resin, and Thymosin synthesized on DEG-PS rink-amide-AM resin. For ABC analogue, 20–60% of mobile phase B in 15 min gradient elution; for Thymosin, 5–30% of mobile phase B in 15 min gradient elution. Experimental conditions 2. Thymosin, H-Ser-Asp-Ala-Ala-Val-Asp-Thr-Ser-Ser-Glu-Ile-Thr-Thr-Lys-Asp-Leu-Lys-Glu-Lys-Lys-Glu-Val-Val-Glu-Glu-Ala-Glu-Asn-NH_2_; ABC analogue, H-Val-Tyr-Trp-Thr-Ser-Pro-Phe-Met-Lys-Leu-Ile-His-Glu-Gln-Cys-Asn-Arg-Ala-Asp-Gly-OH.

The data in Fig. 10 reaffirms the superiority of DEG-PS over DVB-PS. In both peptide syntheses, DEG-PS consistently achieved higher purity (Fig. 11 and S34–S39, ESI Tables 2 and 3†). Despite ChemMatrix resin's ability to deliver difficult peptides with high purity, it is known for its poor peptide yields. For instance, only 26.7% of the ABC analogue peptide and 31.8% of the ABRF 1992 peptide were recovered when synthesized on ChemMatrix resin,^46^ However, DEG-PS yielded nearly double those amounts, with 58.3% recovered for ABC analogue and 56.2% for ABRF 1992. This limitation is a key factor that hampered its widespread adoption in industrial applications, leading to its discontinuation.^5,47^

The isolated crude yields of the lyophilized peptides from DEG-PS resin were higher than those obtained from DVB-PS resin. Moreover, peptides synthesized using the GSPPS method also showed high yields (Fig. 9 and 10).

This trend continued with more complex and longer peptides, such as Thymosin, where DEG-PS still outperformed DVB-PS, achieving purities of 58.4% compared to 54.0% (Fig. 10).

In conclusion, the DEG-PS resin demonstrated enhanced interactions and improved kinetics across multiple SPPS steps, including acylation and cleavage, leading to higher yields and purer final products. Computational studies supported these observations, which were further validated through experimental results.

## 3. Experimental details

### 3.1 Methods and materials

DVB-PS based resin SunResin AM resin (0.70 mmol g^−1^, per the supplier's specifications) and Wang resin (0.56 mmol g^−1^, per the supplier's specifications), DEG-PS based resin SunResin AM resin (0.79 mmol g^−1^, per the supplier's specifications) and Wang resin (0.56 mmol g^−1^, per the supplier's specifications) were utilized for all syntheses. Reagents and solvents were sourced from commercial suppliers and used as received, unless otherwise specified. The mobile phase consisted of 0.1% TFA in H_2_O (Phase A) and 0.1% TFA in CH_3_CN (Phase B), with a flow rate of 1.0 mL min^−1^. The mobile phase consisted of 0.1% formic acid (FA) in H_2_O (Phase A) and 0.1% FA in CH_3_CN (Phase B). A flow rate of 0.4 mL min^−1^ was used with a gradient consisting of 0.5 min of 5% Phase B in Phase A then a 5–95% linear gradient of Phase B over 4 min before a 1 min wash with 95% Phase B and a decrease to 5% Phase B over 0.5 min and further equilibration with 5% Phase B for 0.5 min. High-resolution mass spectra were obtained using positive mode ion detection with a mass window of 50 to 2000 Da. MS^E^ spectra of Thymosin and ABC analogue peptides were obtained using collision energy ramps of 15 V to 30 V, and 30 V to 60 V. Experimental conditions are detailed in Table 2.

**Table 2:** HPLC and LCMS experimental conditions

Attribute	Conditions 1	Conditions 2
**HPLC**		
Machine	Agilent 1260 infinity series system (Agilent Technologies, Waldbronn, Germany) equipped with a G1311B 1260 quaternary pump, G1329B 1260 auto-sampler, vacuum degasser and G1316A 1260 column-controlled compartment	Agilent 1260 infinity II equipped with a G7111B 1260 quaternary pump, G7129A 1260 vial autosampler, and G7121A 1260 fluorescence detector
Column	Symmetry Luna C_18_ (3.6 μm, 4.6 × 150 mm)	Agilent Eclipse XDB-C_18_ 5 μm (4.6 × 150 mm)
Column oven	25 °C	40 °C
Wavelength	220 nm	214 nm

**LCMS**		
Machine	LCMS Agilent Infinity II 1260 UPLC-MSD-XT	UPLC-HRMS, Waters Acquity UPLC-Class I
Column	Thames Restek Raptor C_18_ Column (2.7 μm, 3.0 × 100 mm)	Waters ACQUITY UPLC BEH C_18_, (1.7 μm, 2.1 × 50 mm)
Detector	Electrospray (ESI)	Waters Xevo-G2-XS QTof detector with electrospray ionisation source

### 3.2 Resin synthesis

All SPPS resins were synthesized using suspension polymerization. This involved stirring an organic phase (comprising monomers and initiators) in an aqueous phase (water and stabilizing polymers) at a controlled rpm to form organic droplets with a well-controlled size distribution. The suspension was then heated to 80 °C for several hours, allowing the droplets to harden and form resin beads. These beads were cleaned with hot water and solvents to remove any unreacted chemicals.

The DEG-PS beads contained 3% w/w of DEG monomer, with the remainder being styrene. For the DVB-PS beads, 1% w/w of the monomer mix was a DVB/EVB combination (divinyl benzene : ethyl vinyl benzene = 80 : 20, referred to as DVB), with the rest being styrene. The molar percentage of DEG was 1.31%, while DVB was 0.8%. Despite the lower molar percentage, DEG resins exhibited higher swelling compared to DVB resins, attributed to the molecular differences between the two crosslinkers.

Both DEG-PS and DVB-PS base beads were chloromethylated using the same chemical process. The chloromethylated beads were further functionalized with aminomethyl groups to produce DEG-PS-AM and DVB-PS-AM beads, or with 4-hydroxybenzylalcohol to create DEG-PS-Wang and DVB-PS-Wang SPPS resins for peptide synthesis. After functionalization, the beads were thoroughly cleaned to remove any unreacted compounds and then dried to yield the final SPPS resins.

### 3.3 SEM measurements

The topography of the sputter gold coated resin beads was analyzed using SEM with Zeiss instrumentation.

### 3.4 Swelling experiments

About 200 to 300 mg of resin was placed in a graduated SPPS tube and the corresponding solvent (3.0 mL) was added independently. The resin suspension was left overnight at RT, then excess solvent was removed, and the swollen volume was measured. The swelling degree was calculated using the following equation:
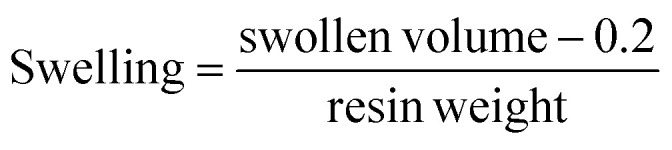
0.2 mL is the occupied volume by the fitted filter in the SPPS tube.

### 3.5 Computational study

To explore the unexpected effects of 2-MeTHF on DEG-PS resin, several simulation tests were performed using BIOVIA Materials Studio 2023 (Accelrys Inc., San Diego, CA, USA).

PS structure was pre-supported within the Materials Studio interface and then crosslinked with either DEG or DVB. The resulting structure contained 1% DEG or DVB with a molecular weight of 22 308.4 g mol^−1^ and 22 023.1 g mol^−1^, respectively. Additionally, the 2-MeTHF assemblies were generated atomistically within the Materials Studio interface for further analysis.

All assemblies underwent energy minimization and geometry optimization using the “Forcite” module with smart algorithms, applying the “Dreiding” forcefield and “Charge using QEq” settings. Swelling capacities were simulated using the “Blends” module, where similar forcefield and charge settings were applied. The calculations focused on predicting *E*_mix_, interaction energy, and the polymer–solvent interaction parameter *χ*.

The computationally obtained *χ* and *E*_mix_ values reflect the degree of miscibility or swelling capacity between the polymer and solvents. Higher *χ* or *E*_mix_ values indicate lower miscibility and swelling capacity, and lower values suggest greater compatibility.

### 3.6 First amino acid anchoring onto resin

#### 3.6.1 Wang resin

Wang resin was swelled in the 2-MeTHF for 10–20 min. Fmoc-amino acid (5 equiv), were dissolved in 2-MeTHF (1.0 mL/100 mg resin), vortexed for 5 min. Then, diisocarbodiimide (DIC) (2.5 equiv.) was added to the amino acid solution. Finally, the mixture and (0.25 equiv.) DMAP were added to the resin. The resulting mixture was allowed to react under mechanical shaking for 2 h at 0 °C then 2 h at RT. The resin was then washed twice with 2-MeTHF (1.0 mL), and the remaining hydroxyl groups were capped with acetic anhydride-DIEA (10 : 20 equiv., respectively). Finally, the resin was washed twice with 2-MeTHF (1.0 mL).

#### 3.6.2 Rink-amide linker anchoring onto aminomethyl (AM) resin

AM resin (200 mg) was swelled in the DMF (1.0 mL) for 10–20 min. Fmoc-Rink-amide linker (2 equiv.), 1-Cyano-2-ethoxy-2-oxoethylidenaminooxy) dimethylamino-morpholino-carbenium hexafluorophosphate (COMU) (1.9 equiv.), were dissolved in DMF (1.0 mL/100 mg resin), vortexed for 5 min. Then, DIEA (4 equiv.) was added to the rink-amide linker and COMU solution. Finally, the cocktail was added to the resin. The resulting mixture was allowed to react under mechanical shaking for 4 h at RT. The resin was then washed twice with DMF (1.0 mL) and twice with 2-MeTHF (1 mL), and the remaining amine groups were capped with acetic anhydride–DIEA (10 : 20 equiv., respectively). Finally, the resin was washed twice with 2-MeTHF (1.0 mL). For this step, 2-MeTHF could not be used due to the insolubility of COMU in this solvent.

#### 3.6.3 Fmoc removal

The Fmoc was removed using 20% piperidine/2-MeTHF and the mixture was allowed to shake for 30 min at 40 °C. The resin was then washed twice with 2-MeTHF.

#### 3.6.4 Peptide synthesis

Peptides were synthesized following the standard methodology performed in our laboratory (3 equiv. of Fmoc-AA-OH, 3 equiv. of OxymaPure, 3 equiv. of DIC) in 2-MeTHF, and then shaking for 1 h at 40 °C. The Fmoc was then removed as per the Fmoc removal section. In the optimization study, the reaction was also conducted at RT for comparison and optimization purposes.

#### 3.6.5 Automated peptide synthesis

Peptides were synthesized on a 0.10 mmol scale using a Biotage Initiator + Alstra peptide synthesizer with DMF solvent. Within the automated SPPS method, amino acid coupling reactions were performed through addition of Fmoc-protected amino acid (0.50 mmol, 5 eq.), OxymaPure (0.50 mmol, 5 eq.), and DIC (1.0 mmol, 10 eq.) to the resin the mixture was subsequently mixed and heated at 90 °C for 2 min. Fmoc-deprotection was performed for 10 min at RT using 20% piperidine in DMF (4.5 mL) with 0.1 M OxymaPure additive. Arginine residues, as well as residues beyond position 20 in the synthesis of the Thymosin peptide, were double coupled to ensure complete incorporation.

#### 3.6.6 Cleavage protocol

Peptides not containing cysteine residues were cleaved from the resin using TFA–triisopropylsilane (TIS)–H_2_O (95 : 2.5 : 2.5) (1 mL/100 mg) under mechanical shaking for 3 h, at RT. Cysteine-containing peptides were cleaved from the resin in the same way using a mixture of TFA/H_2_O/thioanisole/phenol/ethane-1,2-dithiol with a 82.5 : 5 : 5 : 5 : 2.5 ratio (1 mL/100 mg). Chilled diethyl ether was then added (five times the cleavage solution volume), and the solution was kept refrigerated for 30 min. The solution was then centrifuged for 10 min at 5000 rpm, and the supernatant was decanted. Ether (five times the cleavage solution volume) was added to repeat this step. Any remaining ether was removed under N_2_. Finally, the precipitate was dissolved in H_2_O–CH_3_CN (1 : 1). A small amount of the solution was analyzed by HPLC to check the purity of the final product. For the highly hydrophobic peptide, β (34–42), the peptide migrated to the ether layer. To analyze this peptide, the ether layer has been evaporated, and the remaining peptide was dissolved in 50% aqueous acetic acid.

#### 3.6.7 Recovery

The cleaved peptides were lyophilized using a freeze dryer for two days. To calculate the percentage yields, the lyophilized peptides were weighed and compared to the theoretical weight, which was based on the resin loading and the scale of the cleaved peptidyl resin.

## 4. Conclusion

DEG-PS resin offers significant advantages over DVB-PS in peptide synthesis, providing higher yields, better purity, and alignment with green chemistry principles. Its enhanced swelling properties improve reaction kinetics, facilitating the efficient synthesis and cleavage of complex peptides. Green solvents and reagents such as 2-MeTHF, OxymaPure, and COMU were used,^6,48^ with further sustainability improvements achievable by replacing DIC with alternatives like T3P®.^37^ Using the optimized protocol developed in this study, we evaluated several challenging peptide sequences, including β (34–42), JR, and ABRF 1992. DEG-PS consistently produces high peptide yields, outperforming other resins like ChemMatrix, which are known for lower yields.^5,47^ This characteristic is particularly crucial for industrial-scale peptide production, where maximizing yields can significantly reduce overall costs. DEG-PS's reliable performance, even at higher temperatures, makes it ideal for industrial applications, offering cost savings through optimized yields. Future research on its compatibility with other reagents could further confirm its industrial viability.

## Data availability

The authors confirm that the data supporting the findings of this study are available within the article and its ESI.

## Author contributions

Conceptualization: OA, SS; data curation: OAM, JT, CZ; formal analysis: OAM, JT, SMB, CZ; funding acquisition: OAM, SMB; investigation: OAM, JT, CZ; methodology: OAM, JT, SMB; project administration: OAM; software: OAM; resources: OAM, SS, AB, SMB; supervision: OAM, SMB; validation: OAM, SMB; visualization: OAM, JT; writing-original draft: OAM; JT; CZ; writing-review & editing: All authors.

## Conflicts of interest

The authors declare no competing financial interest.

## Supplementary Material

RA-014-D4RA07484J-s001
