# Supplementary material for: Towards green, scalable peptide synthesis: leveraging DEG-crosslinked polystyrene resins to overcome hydrophobicity challenges
Source: RSC Adv. 2024 Dec 23;14(54):40255–66. doi: 10.1039/d4ra07484j (PMC11664329; doi:10.1039/d4ra07484j)
Supplement: RA-014-D4RA07484J-s001 [file RA-014-D4RA07484J-s001.pdf]

Supplementary Information (SI) for RSC Advances.
This journal is © The Royal Society of Chemistry 2024

Towards Green, Scalable Peptide Synthesis: Leveraging DEG-Crosslinked Polystyrene Resins to Overcome Hydrophobicity Challenges

Othman Al Musaimi*[1,2], Joshua Tomkins[3], Sarah M. Barry[3], Alessandra Basso[4], Cheng Zhang[4], Simona Serban*[4]

1. School of Pharmacy, Newcastle University, Newcastle upon Tyne, NE1 7RU, UK

2. Department of Chemical Engineering, Imperial College London, London SW7 2AZ, UK

3. Department of Chemistry, King's College London, Britannia House, 7 Trinity Street, London SE1 1DB, U.K.

4. Life Science Division, Sunresin New Materials Co. Ltd., 710076 Xi'an, China

* Corresponding authors: OAM: othman.almusaimi@newcastle.ac.uk, SS: simona.serban@sunresin.com

Supplementary Information

Index

Supplemental Table 1. Peptides investigated in this study.

Supplementary Table 2: Fragment ions identified within the MS^E spectra obtained for the crude ABC analogue peptide.

Supplementary Table 3: Fragment ions identified within the MS^E spectra obtained for the crude Thymosin peptide.

Supplementary Figure 1. Free energy of DEG-PS.

Supplementary Figure 2. Free energy of DVB-PS.

Supplementary Figure 3. Mass spectrum of Leu-Enkephalin peptide on DVB-PS using DMF at RT.

Supplementary Figure 4. Mass spectrum of double-incorporated Leu of the Leu-Enkephalin synthesis on DVB-PS using DMF at RT.

Supplementary Figure 5. Mass spectrum of Des-Gly-Gly in the crude double-incorporated Leu of the Leu-Enkephalin synthesis on DVB-PS using DMF at RT.

Supplementary Figure 6. Mass spectrum of crude Leu-Enkephalin peptide on DEG-PS using DMF at RT.

Supplementary Figure 7. Mass spectrum of Des-Ile from crude ACP synthesis on DVB-PS using DMF at RT.

Supplementary Figure 8. Mass spectrum of Des-Ala from crude ACP synthesis on DVB-PS using DMF at RT.

Supplementary Figure 9. Mass spectrum of crude ACP peptide on DVB-PS using DMF at RT.

Supplementary Figure 10. Mass spectrum of crude ACP peptide on DEG-PS using DMF at RT.

Supplementary Figure 11. Mass spectrum of Des-Asp in crude ACP synthesis on DVB-PS using DMF at RT.

Supplementary Figure 12. Mass spectrum of Des-Tyr-Ile in crude ACP synthesis on DEG-PS using DMF at RT.

Supplementary Figure 13. Mass spectrum of Des-Ala in crude ACP synthesis on DEG-PS using DMF at RT.

Supplementary Figure 14. Mass spectrum of Des-Ala-Asp in crude ACP crude synthesis on DEG-PS using DMF at RT.

Supplementary Figure 15. HPLC chromatogram (220 nm) of crude ACP peptide synthesis on DVB-PS Wang resin, in 2-MeTHF, at RT.

Supplementary Figure 16. HPLC chromatogram (220 nm) of crude ACP peptide synthesis on DEG-PS Wang resin, in 2-MeTHF, at RT.

Supplementary Figure 17. HPLC chromatogram (220 nm) of crude ACP peptide on DVB-PS Wang resin, in 2-MeTHF, at 40 °C.

Supplementary Figure 18. HPLC chromatogram (220 nm) of crude ACP peptide on DEG-PS Wang resin, in 2-MeTHF, at 40 °C.

Supplementary Figure 19. HPLC chromatogram (220 nm) of crude Aib-ACP peptide on DVB-PS Wang resin, in 2-MeTHF, at RT.

Supplementary Figure 20. HPLC chromatogram (220 nm) of crude Aib-ACP peptide on DEG-PS Wang resin, in 2-MeTHF, at RT.

Supplementary Figure 21. HPLC chromatogram (220 nm) of crude Aib-ACP peptide on DVB-PS Wang resin, in 2-MeTHF, at 40 °C.

Supplementary Figure 22. HPLC chromatogram (220 nm) of crude Aib-ACP peptide on DEG-PS Wang resin, in 2-MeTHF, at 40 °C.

Supplementary Figure 23. Mass spectrum of crude Aib-ACP peptide on DVB-PS using DMF at RT.

Supplementary Figure 24. Mass spectrum of crude Aib-ACP peptide on DEG-PS using DMF at RT.

Supplementary Figure 25. Mass spectrum of crude β (34–42) peptide on DEG-PS using 2-MeTHF at 40 °C.

Supplementary Figure 26. Mass spectrum of crude Fmoc-β (34–42) peptide on DEG-PS using 2-MeTHF at 40 °C.

Supplementary Figure 27. Mass spectrum of crude JR peptide on DEG-PS using 2-MeTHF at 40 °C.

Supplementary Figure 28. Mass spectrum of des-Trp in crude JR peptide on DEG-PS using 2-MeTHF at 40 °C.

Supplementary Figure 29. Mass spectrum of des-Phe in crude JR peptide on DEG-PS using 2-MeTHF at 40 °C.

Supplementary Figure 30. Mass spectrum of des-Thr in crude JR peptide on DEG-PS using 2-MeTHF at 40 °C.

Supplementary Figure 31. Mass spectrum of des-Trp-Thr in crude JR peptide on DEG-PS using 2-MeTHF at 40°C.

Supplementary Figure 32. Mass spectrum of crude Fmoc-JR peptide on DEG-PS using 2-MeTHF at 40°C.

Supplementary Figure 33. Mass spectrum of crude ABRF1992 peptide on DEG-PS using 2-MeTHF at 40°C.

Supplementary Figure 34. High resolution mass spectrum of crude ABC analogue peptide on DEG-PS resin.
Supplementary Figure 35. High resolution mass spectrum of crude ABC analogue peptide on DVB-PS resin.
Supplementary Figure 36. MS^E spectra of ABC analogue peptide on DEG-PS resin
Supplementary Figure 37. High resolution mass spectrum of crude Thymosin peptide on DEG-PS resin.
Supplementary Figure 38. High resolution mass spectrum of crude Thymosin peptide on DVB-PS resin.
Supplementary Figure 39. MS^E spectra of Thymosin peptide on DEG-PS resin

Supplemental Tables

Supplemental Table 1. Peptides investigated in this study.

Peptide	Sequence	Formula	Mass
Leu-Enkephalin	H-Tyr-Gly-Gly-Ohe-Leu-OH	$C_{28}H_{37}N_5O_7$	555.27
β (34-42)	H-Leu-Met-Val-Gly-Gly-Val-Val-Ile-Ala-OH	$C_{39}H_{71}N_9O_{10}S$	858.11
ACP	H-Val-Gln-Ala-Ala-Ile-Asp-Tyr-Ile-Asn-Gly-OH	$C_{47}H_{74}N_{12}O_{16}$	1063.18
Aib-ACP	H-Val-Gln-Aib-Aib-Ile-Asp-Tyr-Ile-Asn-Gly-OH	$C_{49}H_{78}N_{12}O_{16}$	1091.23
JR	H-Trp-Phe-Thr-Thr-Leu-Ile-Ser-Thr-Ile-Met-OH	$C_{58}H_{89}N_{11}O_{15}S$	1212.47
ABRF 1992	H-Gly-Val-Arg-Gly-Asp-Lys-Gly-Asn-Pro-Gly-Trp-Pro-Gly-Ala-Pro-Tyr-NH_2	$C_{73}H_{106}N_{22}O_{21}$	1627.78
ABC	H-Val-Tyr-Trp-Thr-Ser-Pro-Phe-Met-Lys-Leu-Ile-His-Glu-Gln-Cys-Asn-Arg-Ala-Asp-Gln-OH	$C_{110}H_{164}N_{30}O_{31}S_2$	2466.82
Thymosin	H-Ser-Asp-Ala-Ala-Val-Asp-Thr-Ser-Ser-Glu-Ile-Thr-Thr-Lys-Asp-Leu-Lys-Glu-Lys-Lys-Glu-Val-Val-Glu-Glu-Ala-Glu-Asn-NH_2	$C_{127}H_{213}N_{33}O_{54}$	3066.28

Supplemental Figures

A

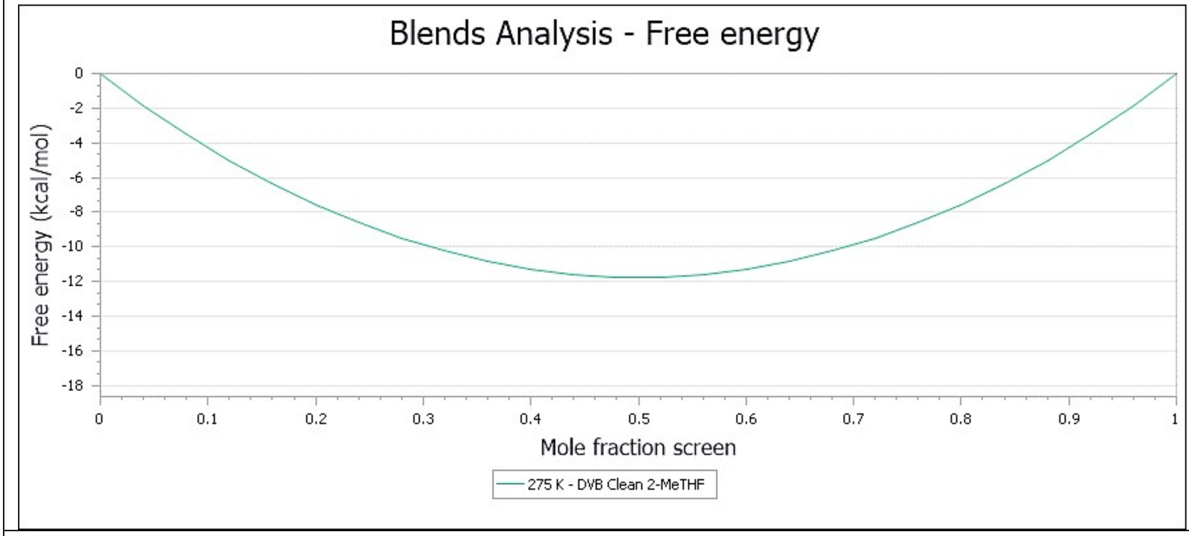

B

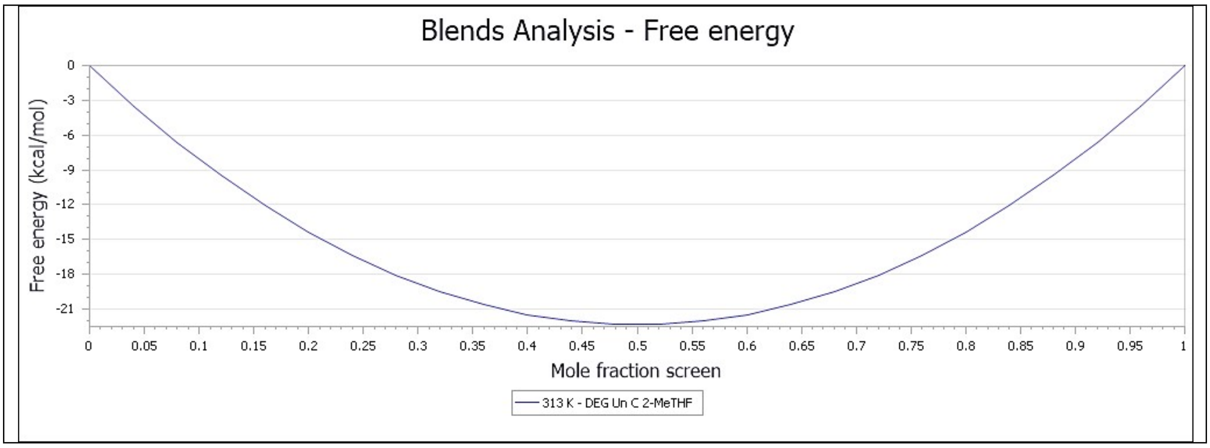

Supplementary Figure 1. Free energy of DEG-PS. A. at RT. B. At 40 ^{o}C.

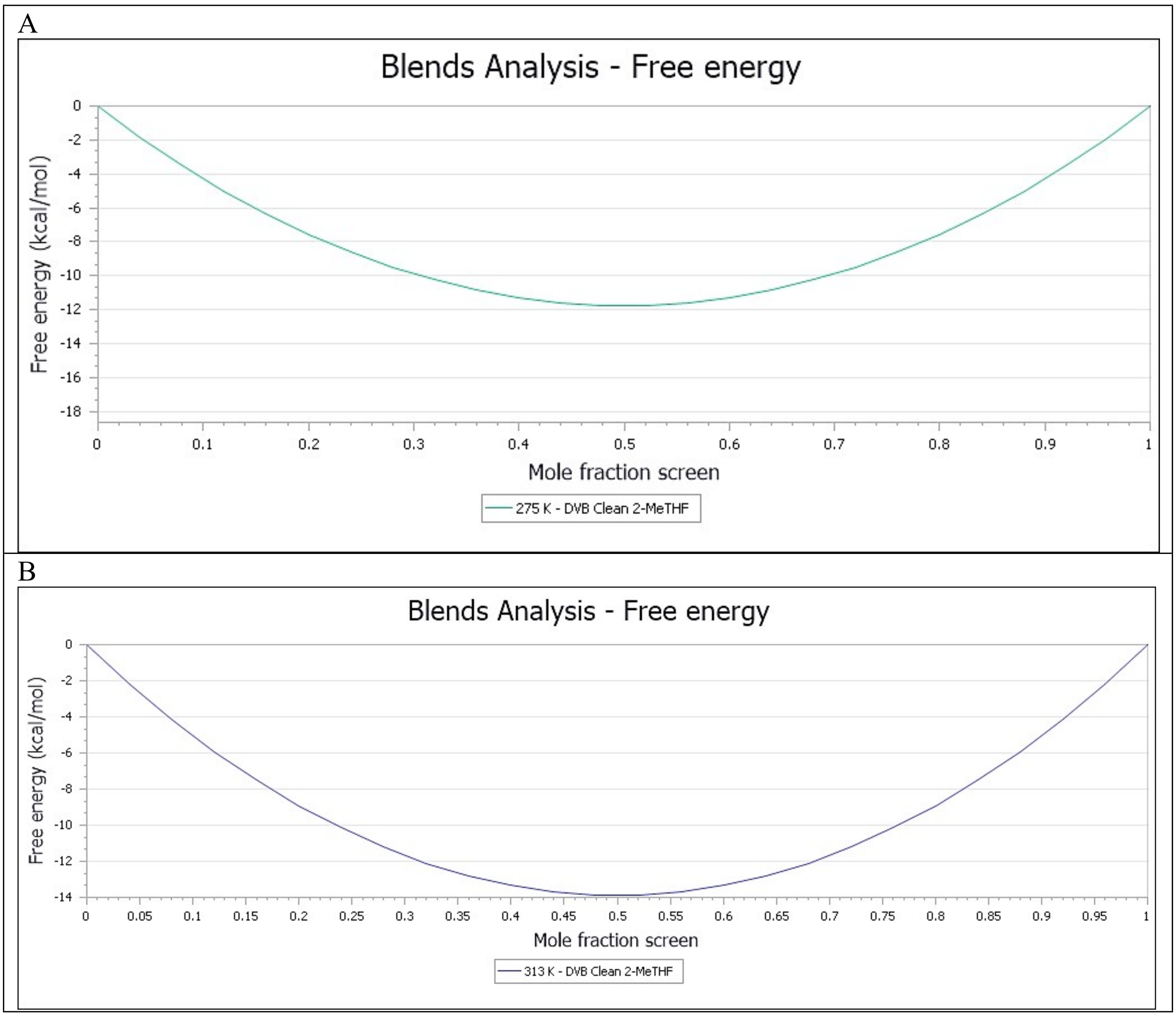

Supplementary Figure 2. Free energy of DVB-PS. A. at RT. B. At 40 ^{o}C.

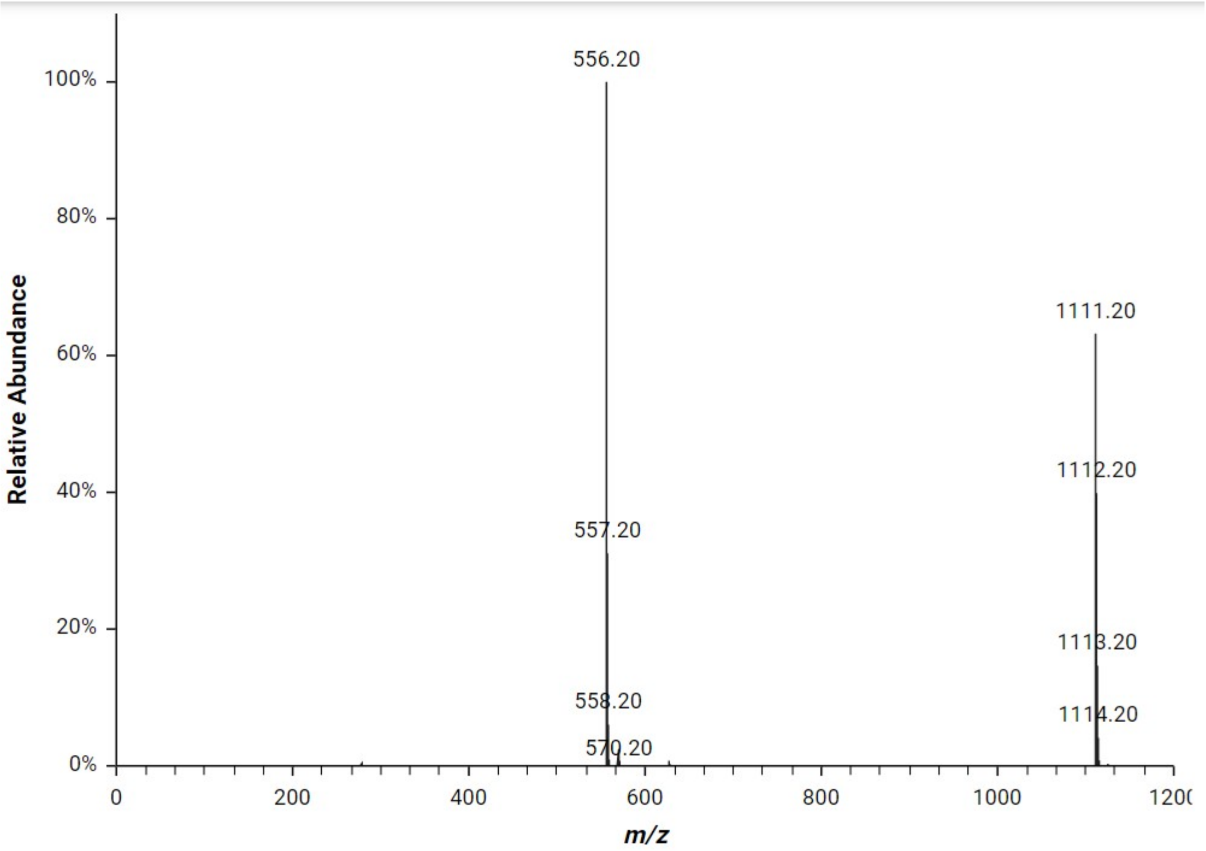

Supplementary Figure 3. Mass spectrum of crude Leu-Enkephalin peptide on DVB-PS using DMF at RT. Calculated for $C_{28}H_{37}N_5O_7$: 555.6 Da, found: 556.2 $[M + H]^+$, 1111.2 $[2M + H]^+$ (non-covalent dimer). Experimental conditions 1.

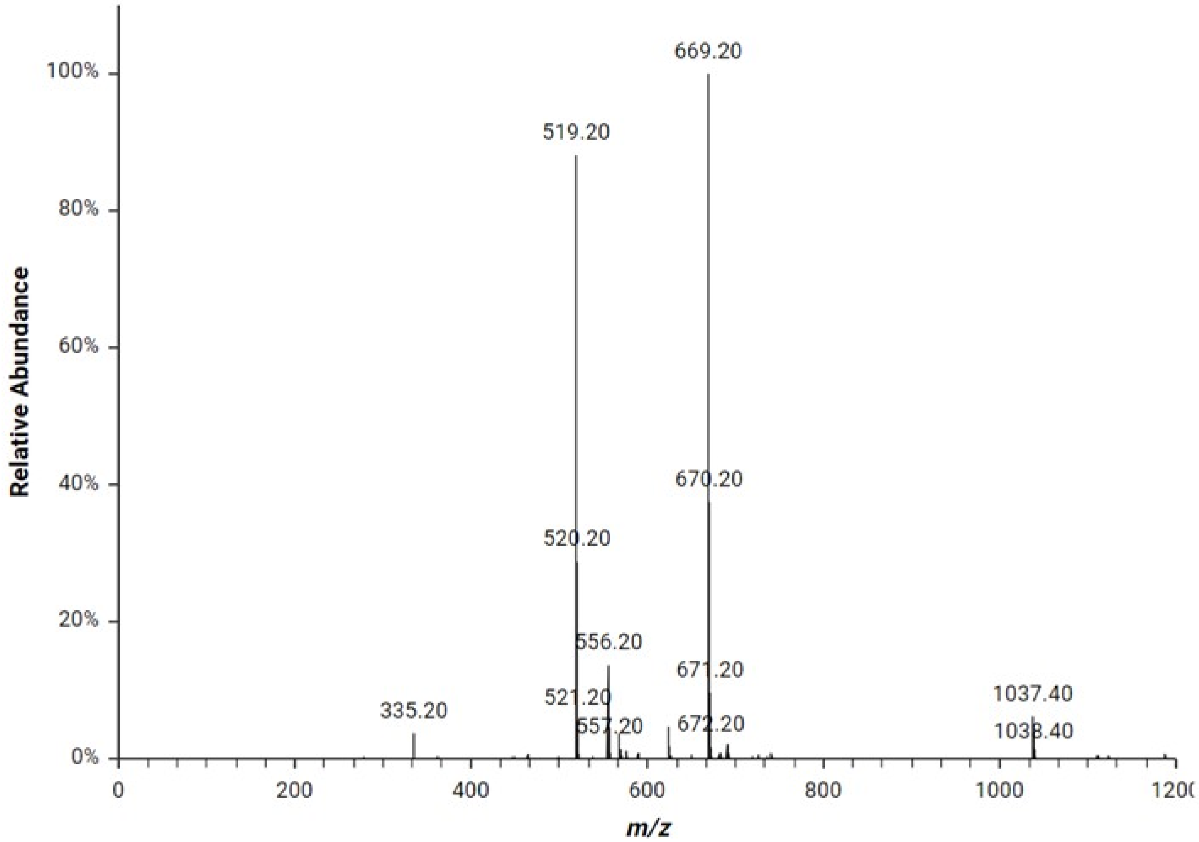

Supplementary Figure 4. Mass spectrum of crude double-incorporated Leu of the Leu-Enkephalin synthesis on DVB-PS using DMF at RT. Calculated for $C_{34}H_{48}N_6O_8$: 668.8 Da, found: 669.2 $[M + H]^+$, 1037.4 $[2M + H]^{2+}$ (non-covalent dimer). Experimental conditions 1.

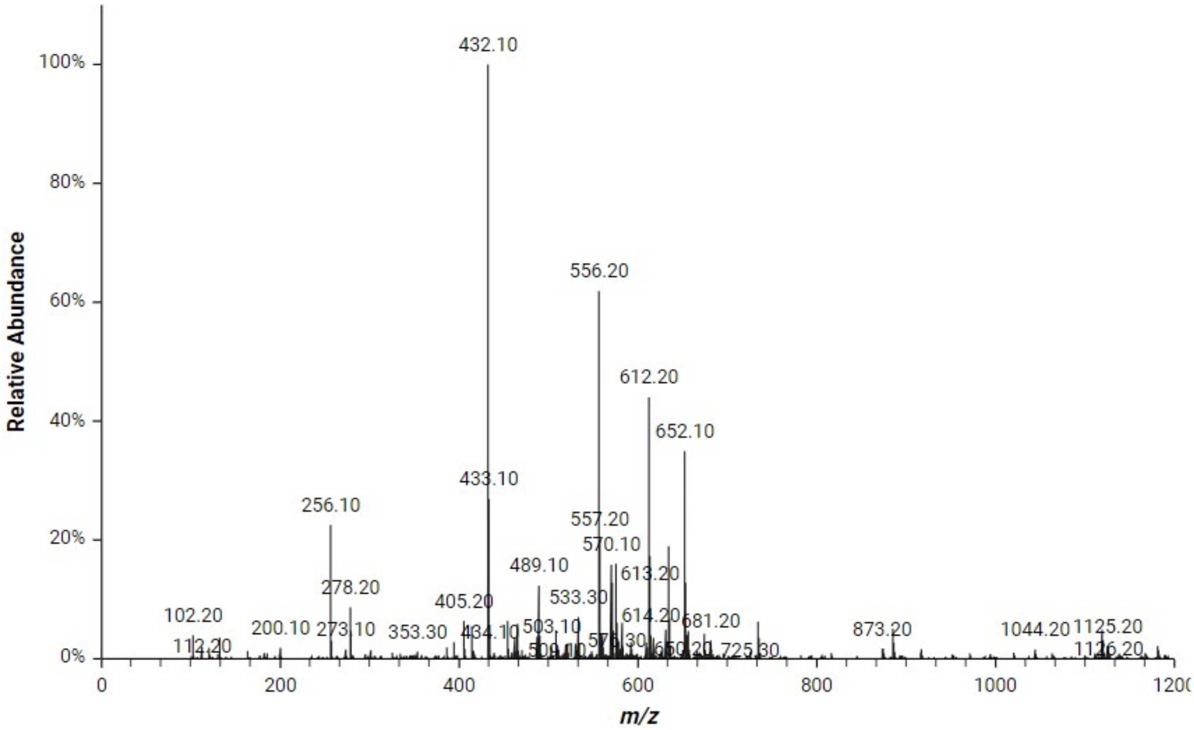

Supplementary Figure 5. Mass spectrum of Des-Gly-Gly in the crude double-incorporated Leu of the Leu-Enkephalin synthesis on DVB-PS using DMF at RT. Calculated for $C_{30}H_{42}N_4O_6$: 554.7 Da, found: 556.2 $[M + H]^+$. Experimental conditions 1.

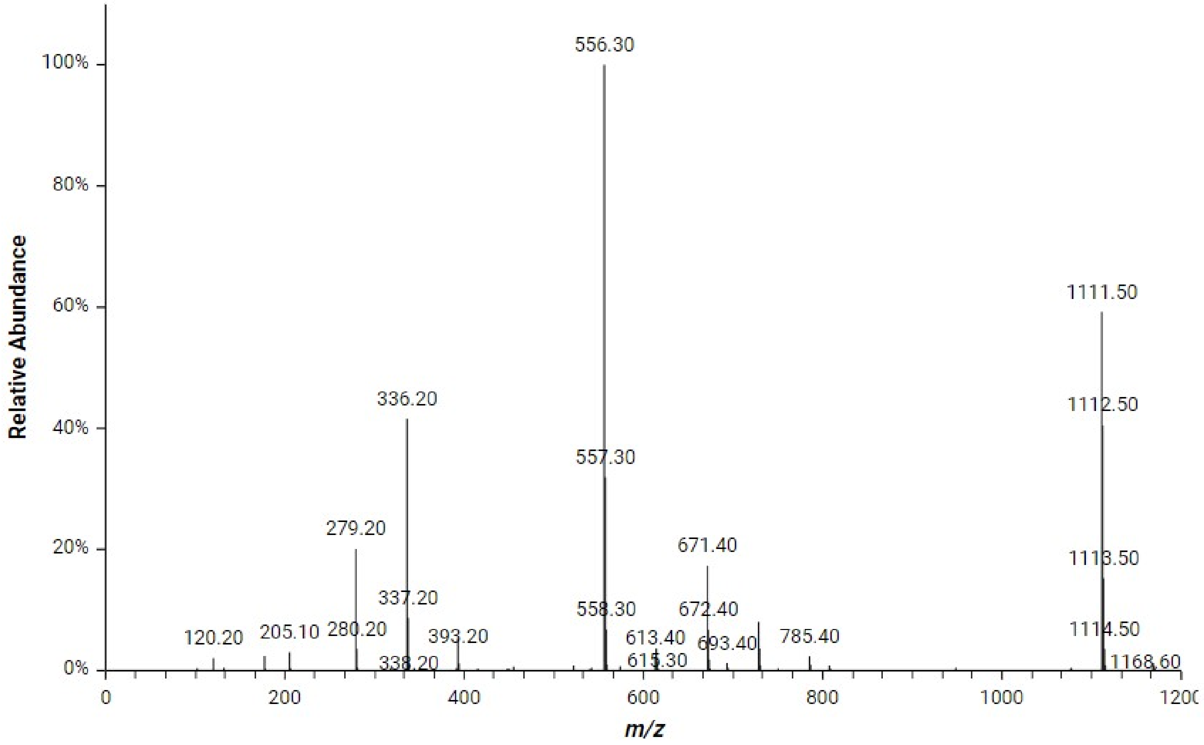

Supplementary Figure 6. Mass spectrum of crude Leu-Enkephalin peptide on DEG-PS using DMF at RT. Calculated for $C_{28}H_{37}N_5O_7$: 555.3 Da, found: 556.2 $[M + H]^+$, 1111.5 $[2M + H]^+$ (non-covalent dimer). Experimental conditions 1.

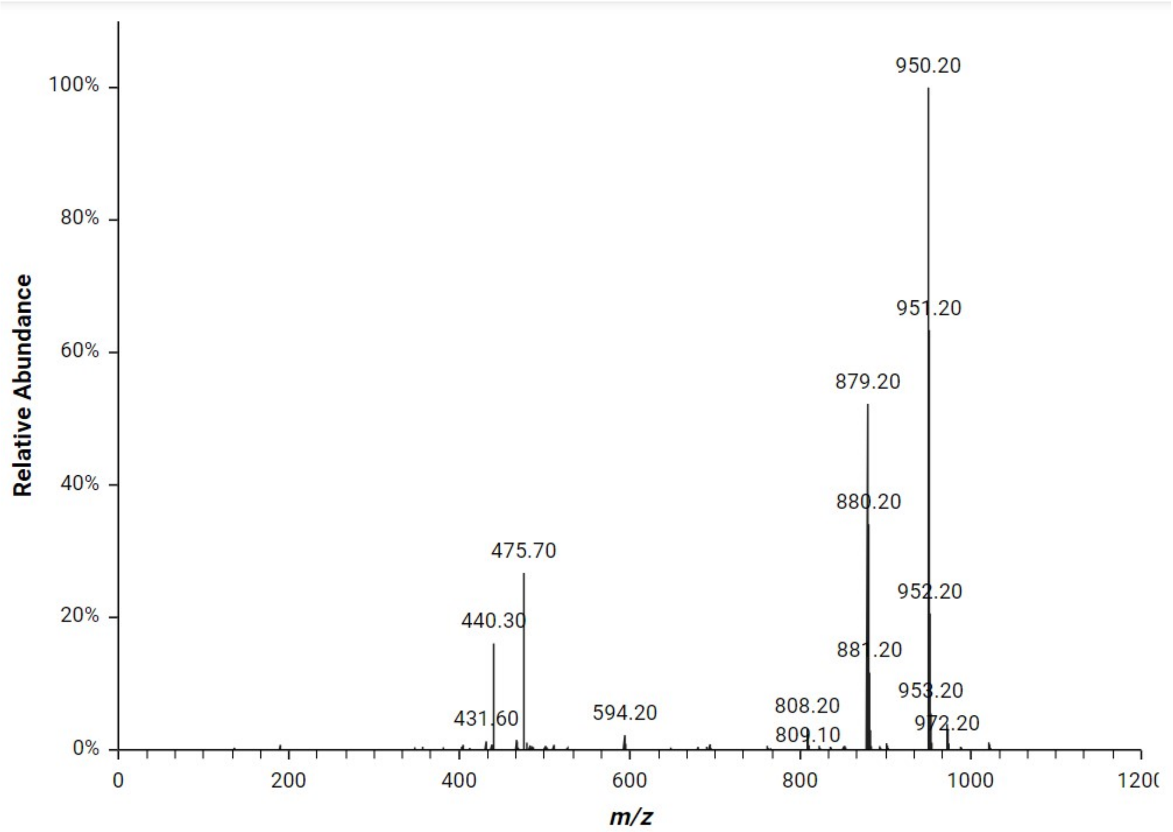

Supplementary Figure 7. Mass spectrum of Des-Ile in crude ACP synthesis on DVB-PS using DMF at RT. Calculated for $C_{47}H_{74}N_{12}O_{16}$: 950.0 Da, found: 951.2 $[M + H]^{+}$, 475.7 $[M + 2H]^{2+}$. Experimental conditions 1.

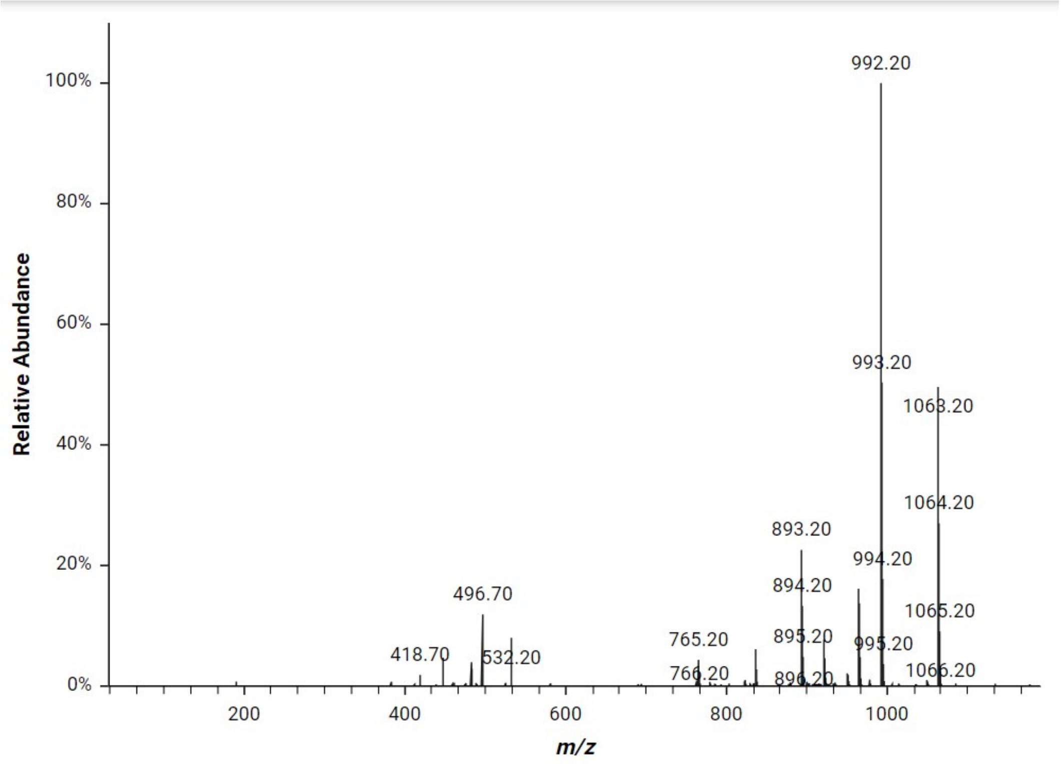

Supplementary Figure 8. Mass spectrum of Des-Ala in crude ACP synthesis on DVB-PS using DMF at RT. Calculated for $C_{44}H_{69}N_{11}O_{15}$: 992.1 Da, found: 993.2 $[M + H]^{+}$. Experimental conditions 1.

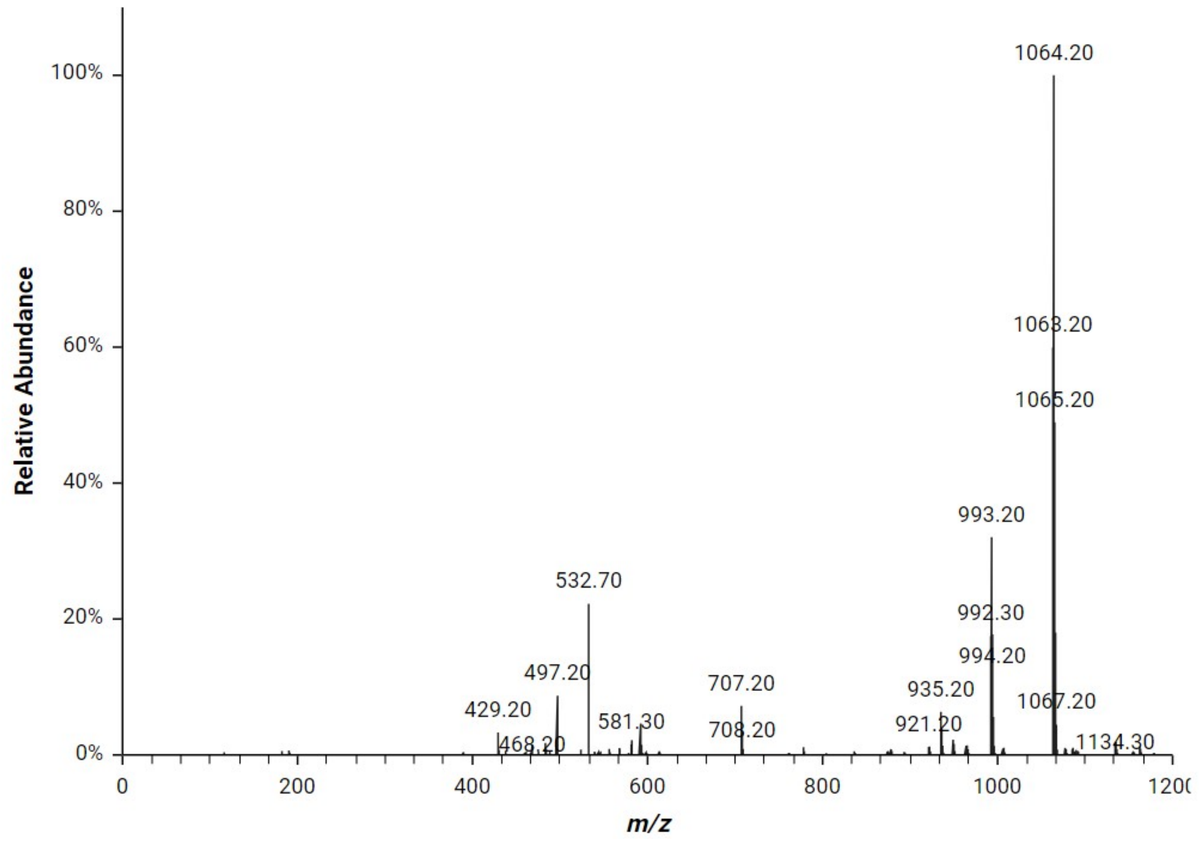

Supplementary Figure 9. Mass spectrum of crude ACP peptide on DVB-PS using DMF at RT. Calculated for $C_{47}H_{74}N_{12}O_{16}$: 1063.2 Da, found: 1064.2 $[M + H]^{+}$, 532.7 $[M + 2H]^{2+}$. Experimental conditions 1.

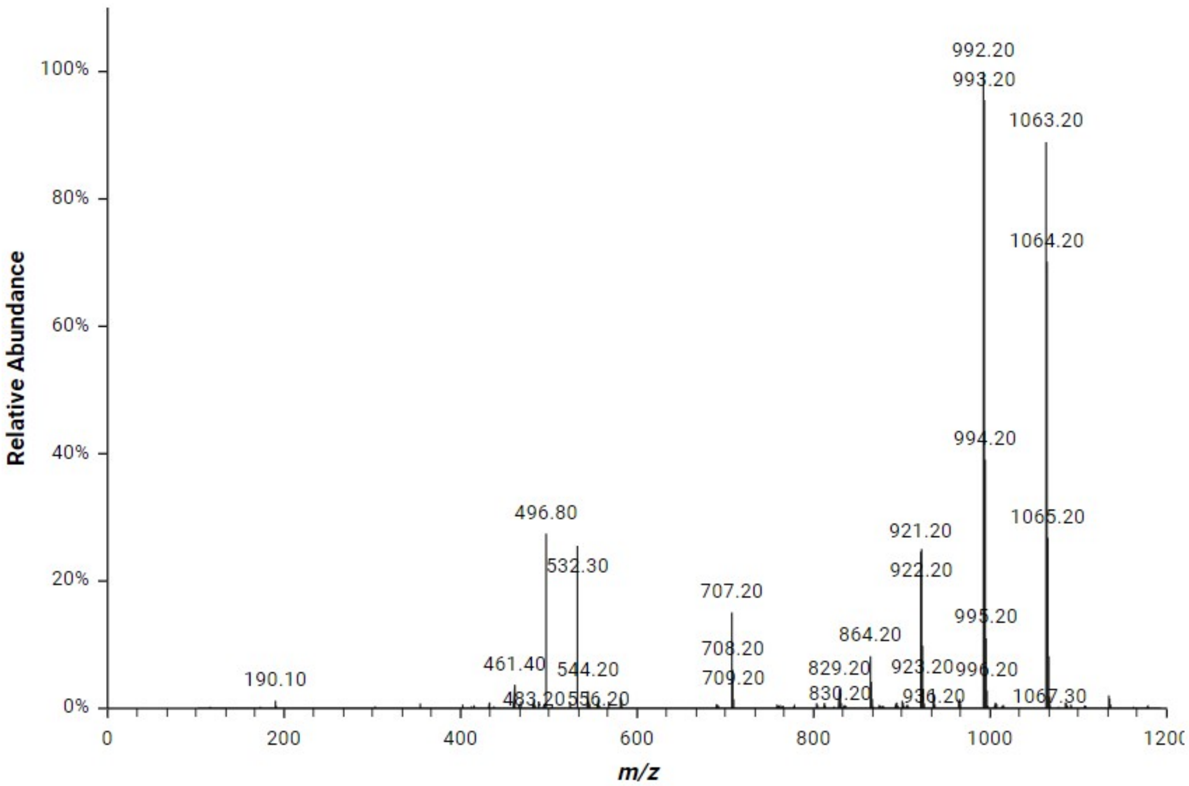

Supplementary Figure 10. Mass spectrum of crude ACP peptide on DEG-PS using DMF at RT. Calculated for $C_{47}H_{74}N_{12}O_{16}$: 1063.2 Da, found: 1064.2 $[M + H]^+$, 532.3 $[M + 2H]^{2+}$. Experimental conditions 1.

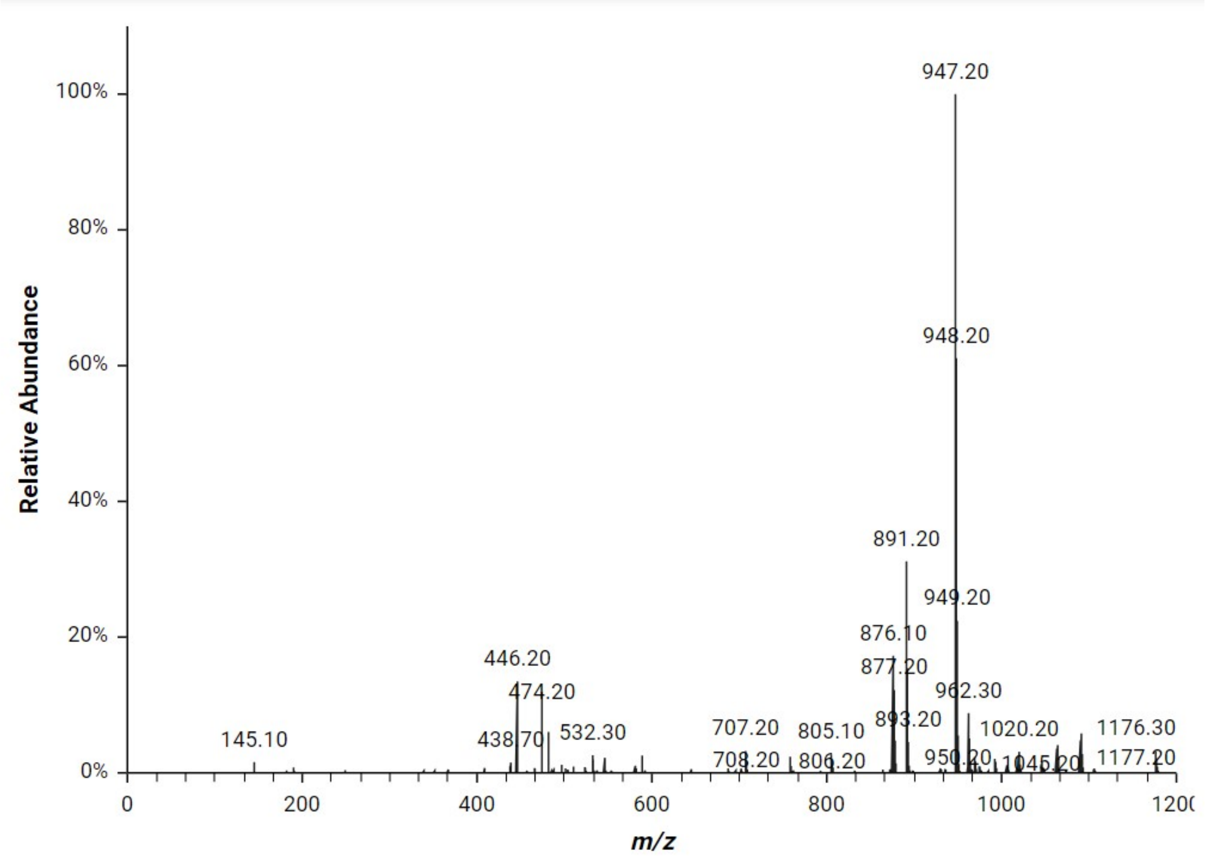

Supplementary Figure 11. Mass spectrum of Des-Asp in crude ACP synthesis on DVB-PS using DMF at RT. Calculated for $C_{43}H_{69}N_{11}O_{13}$: 948.1 Da, found: 949.2 $[M + H]^+$. Experimental conditions 1.

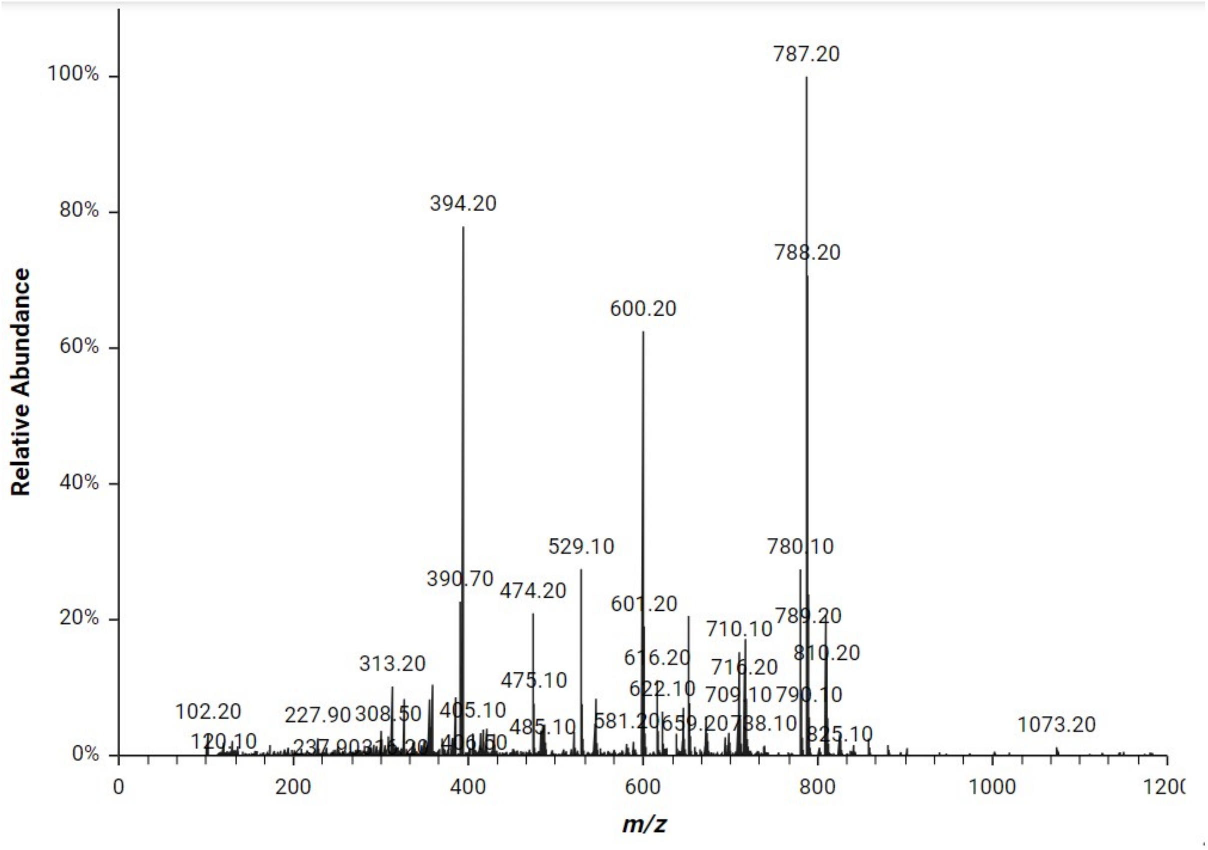

Supplementary Figure 12. Mass spectrum of Des-Tyr-Ile in crude ACP synthesis on DEG-PS using DMF at RT. Calculated for $C_{32}H_{54}N_{10}O_{13}$: 786.8 Da, found: 787.2 $[M + H]^{+}$, 394.2 $[M + 2H]^{2+}$. Experimental conditions 1.

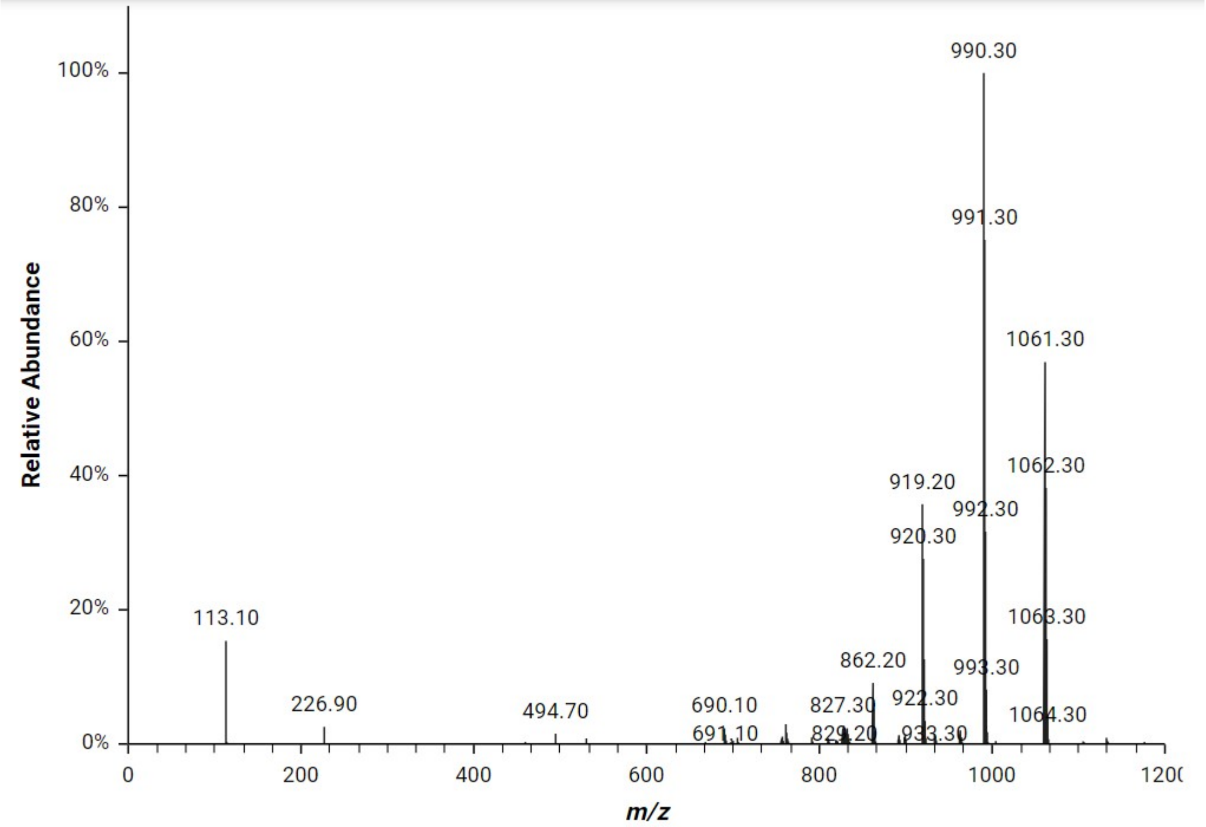

Supplementary Figure 13. Mass spectrum of Des-Ala in crude ACP synthesis on DEG-PS using DMF at RT. Calculated for $C_{44}H_{69}N_{11}O_{15}$: 992.1 Da, found: 993.3 $[M + H]^+$. Experimental conditions 1.

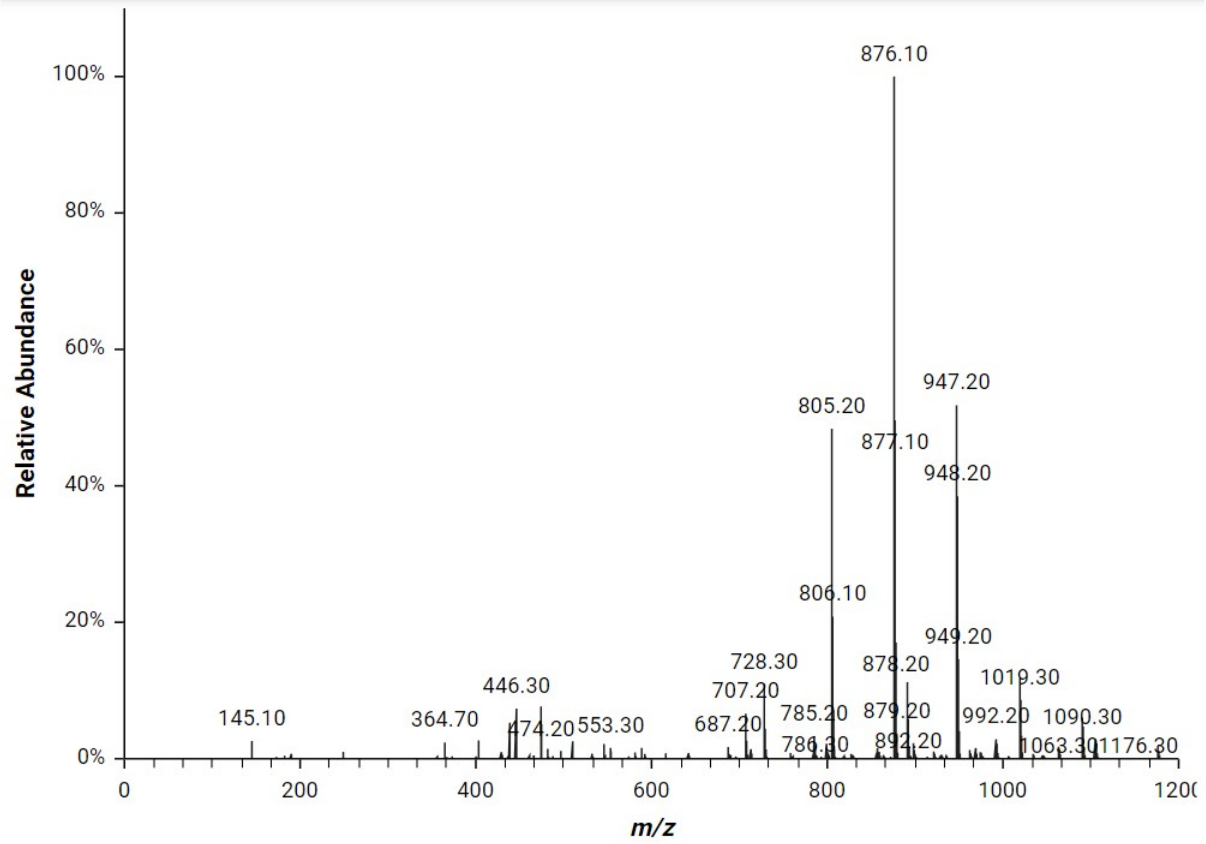

Supplementary Figure 14. Mass spectrum of Des-Ala-Asp in crude ACP crude synthesis on DEG-PS using DMF at RT. Calculated for $C_{40}H_{64}N_{10}O_{12}$: 877.0 Da, found: 878.2 $[M + H]^{+}$. Experimental conditions 1.

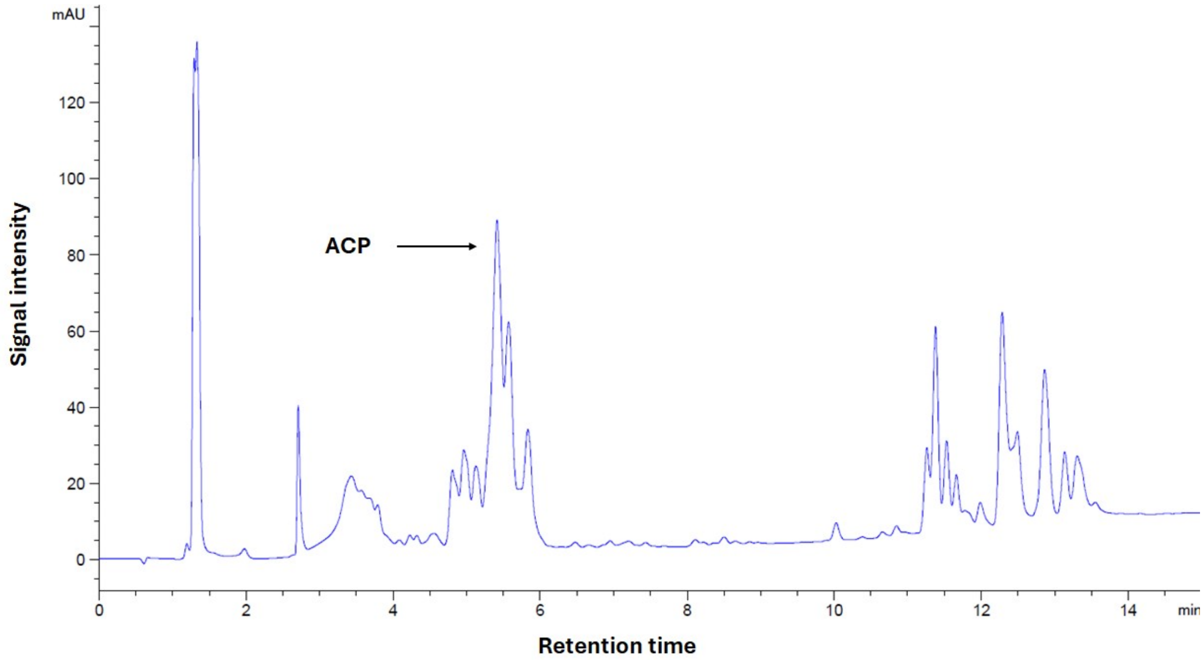

Supplementary Figure 15. HPLC chromatogram (220 nm) of crude ACP peptide synthesis on DVB-PS Wang resin, in 2-MeTHF, at RT. Refer to legend of Figure 8 for chromatographic conditions. Experimental conditions 1.

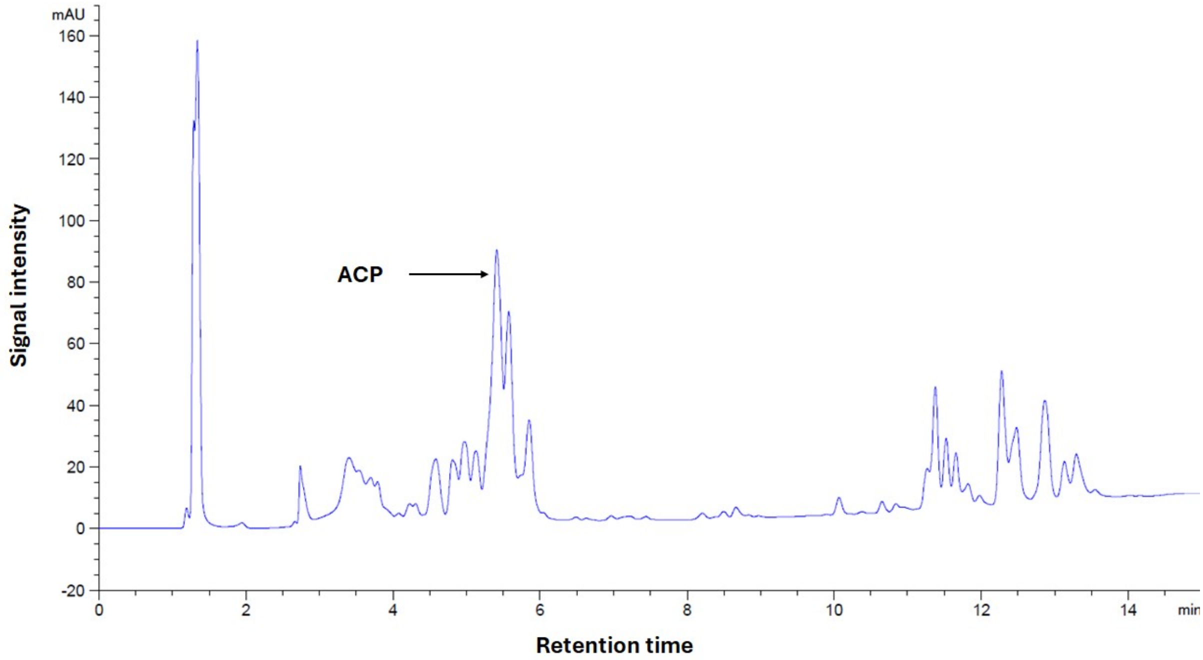

Supplementary Figure 16. HPLC chromatogram (220 nm) of crude ACP peptide synthesis on DEG-PS Wang resin, in 2-MeTHF, at RT. Experimental conditions 1.

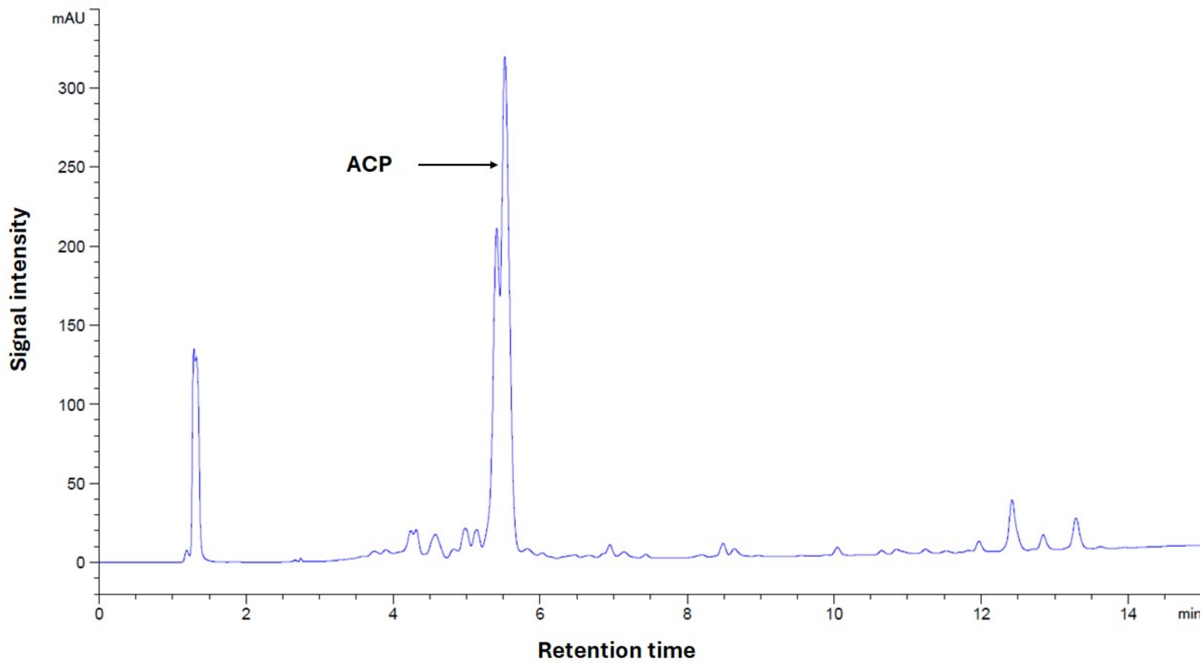

Supplementary Figure 17. HPLC chromatogram (220 nm) of crude ACP peptide on DVB-PS Wang resin, in 2-MeTHF, at 40 °C. Experimental conditions 1.

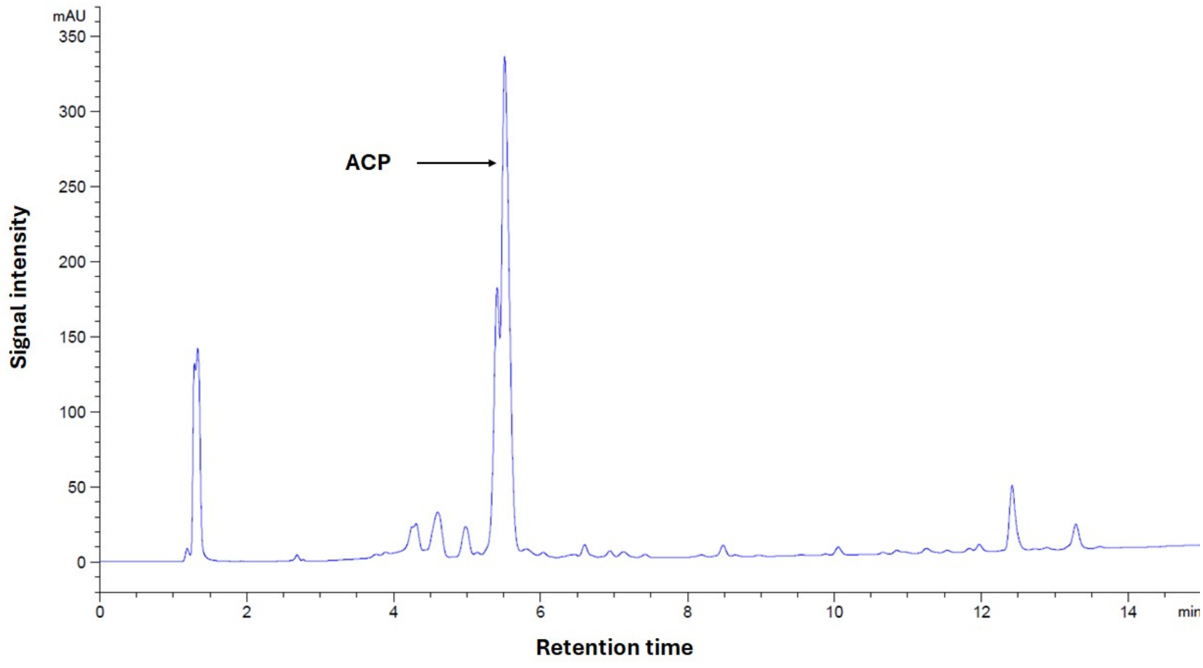

Supplementary Figure 18. HPLC chromatogram (220 nm) of crude ACP peptide on DEG-PS Wang resin, in 2-MeTHF, at 40 °C. Experimental conditions 1.

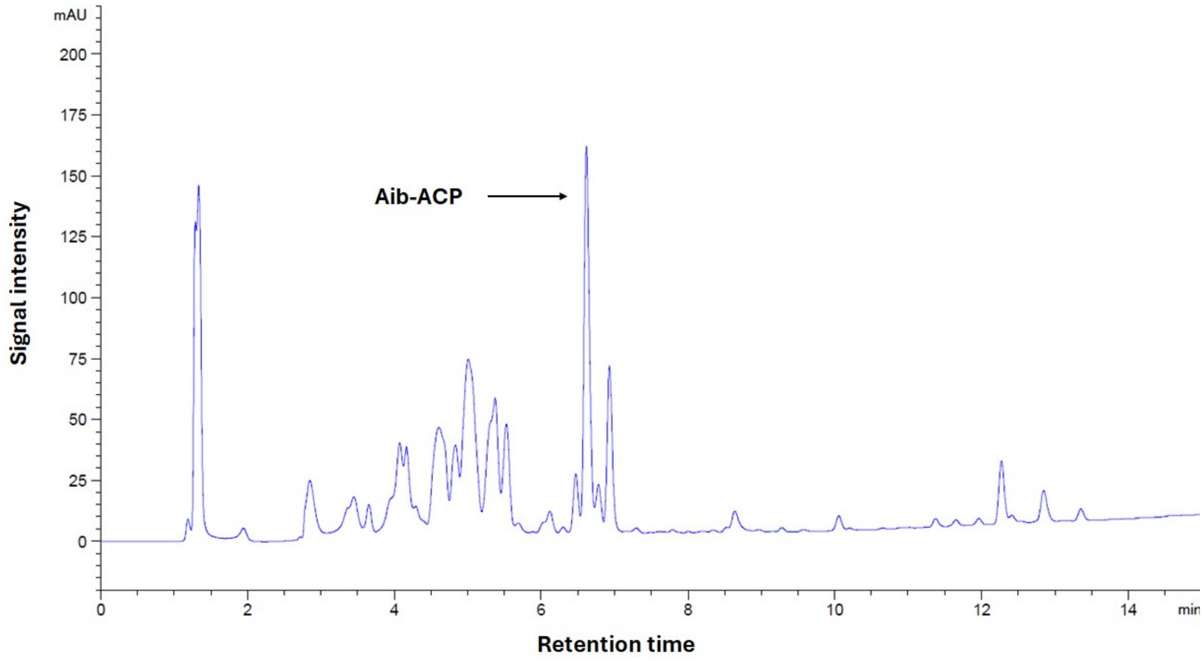

Supplementary Figure 19. HPLC chromatogram (220 nm) of crude Aib-ACP peptide on DVB-PS Wang resin, in 2-MeTHF, at RT. Experimental conditions 1.

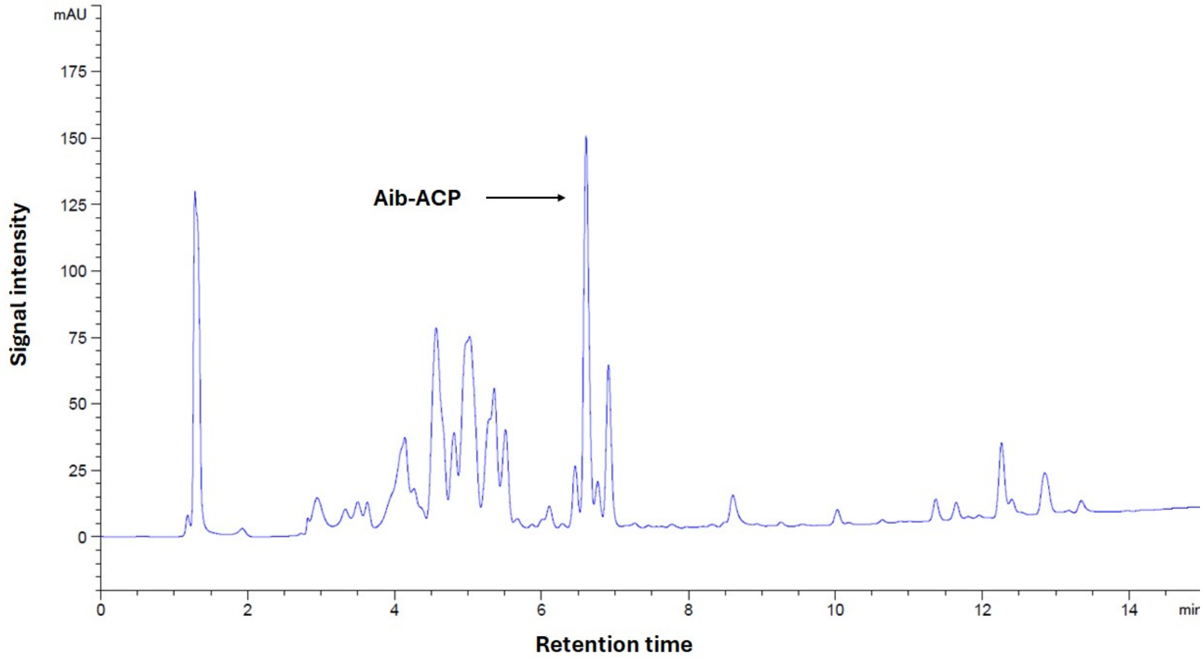

Supplementary Figure 20. HPLC chromatogram (220 nm) of crude Aib-ACP peptide on DEG-PS Wang resin, in 2-MeTHF, at RT. Experimental conditions 1.

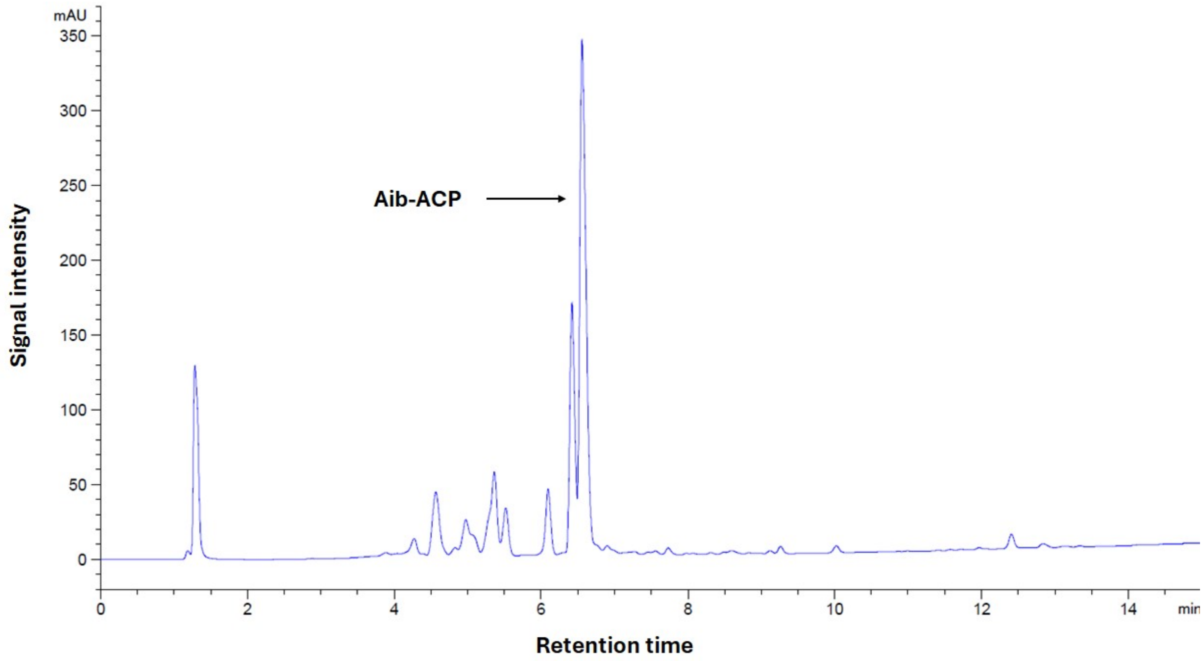

Supplementary Figure 21. HPLC chromatogram (220 nm) of crude Aib-ACP peptide on DVB-PS Wang resin, in 2-MeTHF, at 40 ^{o}C. Experimental conditions 1.

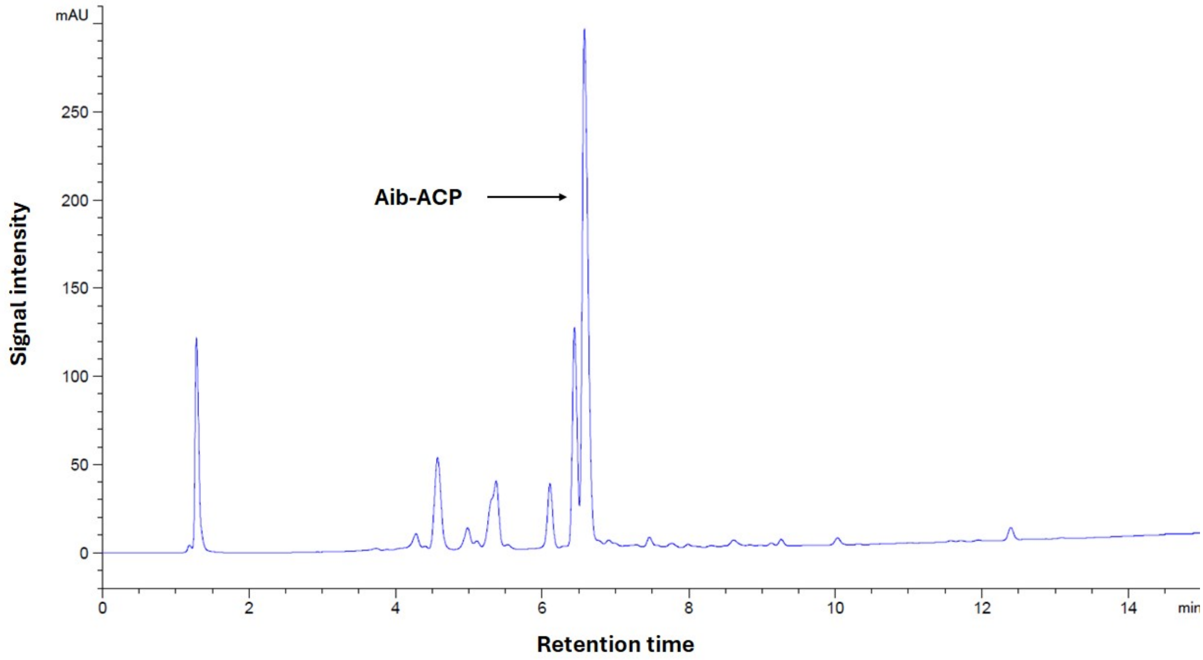

Supplementary Figure 22. HPLC chromatogram (220 nm) of crude Aib-ACP peptide on DEG-PS Wang resin, in 2-MeTHF, at 40 ^{o}C. Experimental conditions 1.

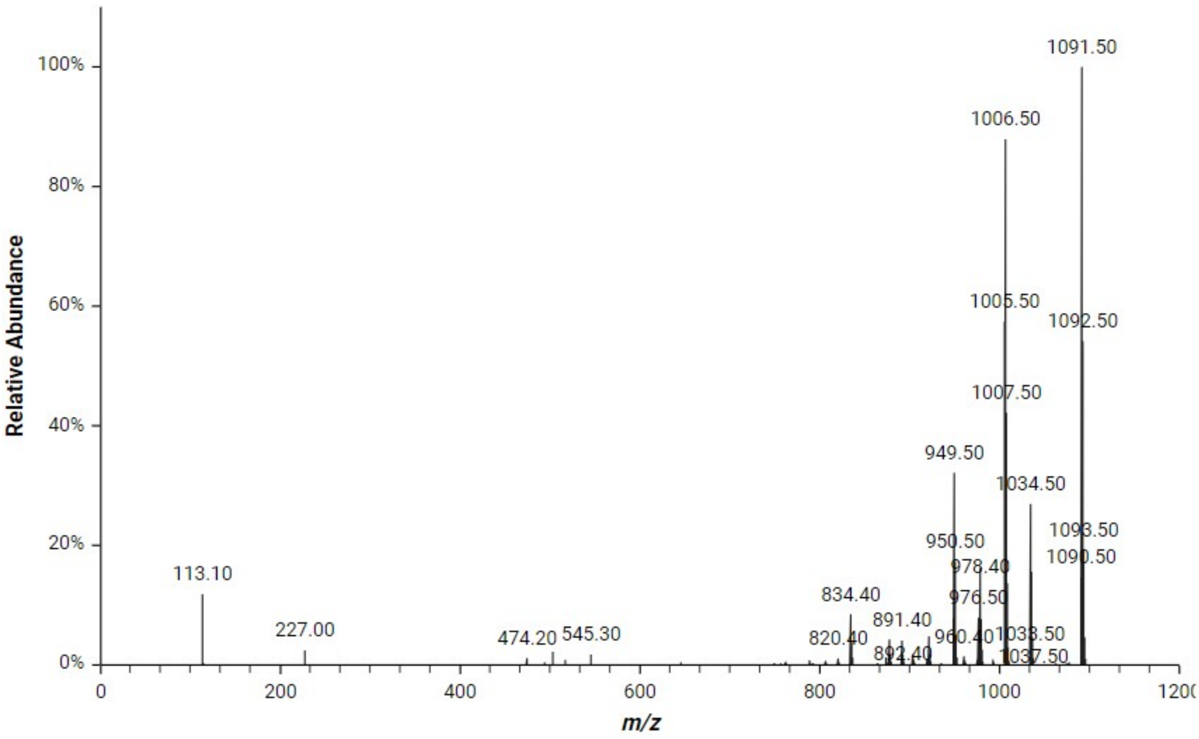

Supplementary Figure 23. Mass spectrum of crude Aib-ACP peptide on DVB-PS using DMF at RT. Calculated for $C_{49}H_{83}N_{12}O_{16}$: 1091.2 Da, found: 1092.5 $[M + H]^+$. Des-Aib, calculated: 1006.1, found: 1006.5 $[M + H]^+$. Experimental conditions 1.

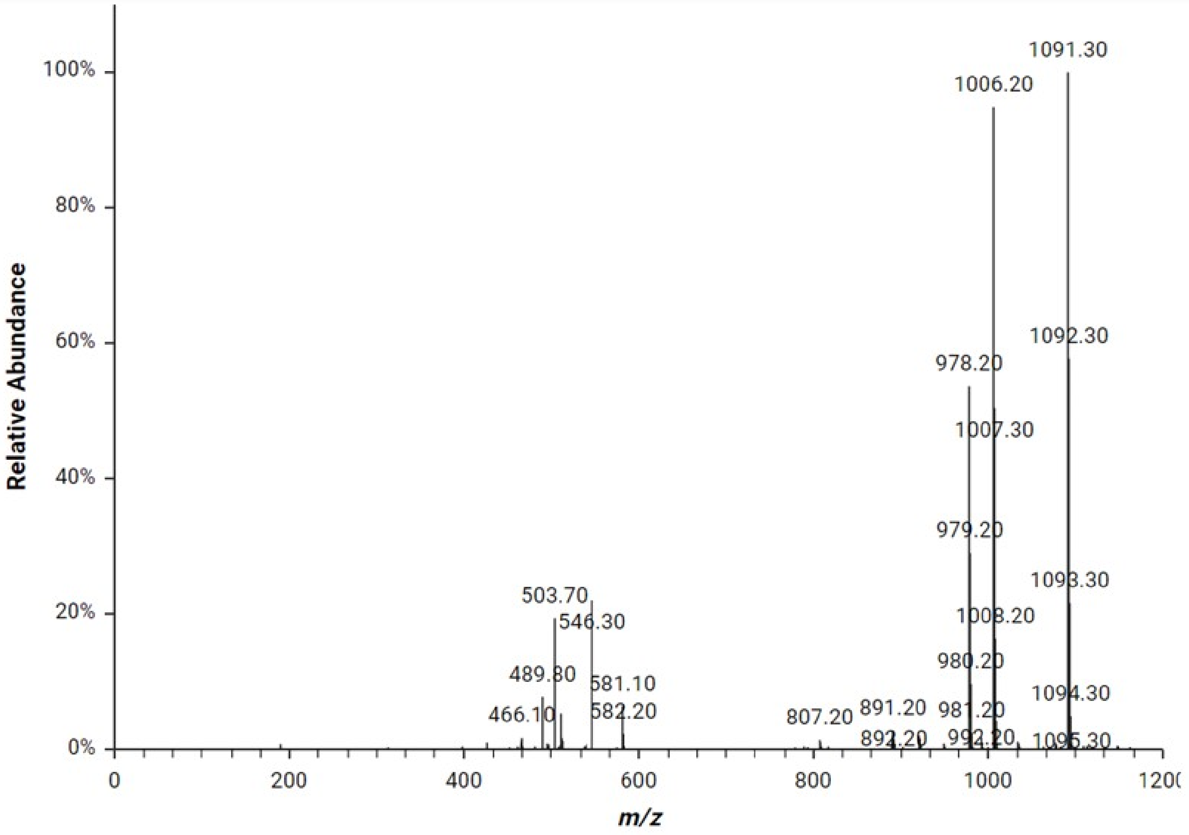

Supplementary Figure 24. Mass spectrum of crude Aib-ACP peptide on DEG-PS using DMF at RT.

Calculated for $C_{49}H_{83}N_{12}O_{16}$: 1091.2 Da, found: 1092.3 $[M + H]^+$, 546.3 $[M + 2H]^{2+}$. Des-Aib, calculated: 1006.1, found: 1007.3 $[M + H]^+$, 503.7 $[M + 2H]^{2+}$. Experimental conditions 1.

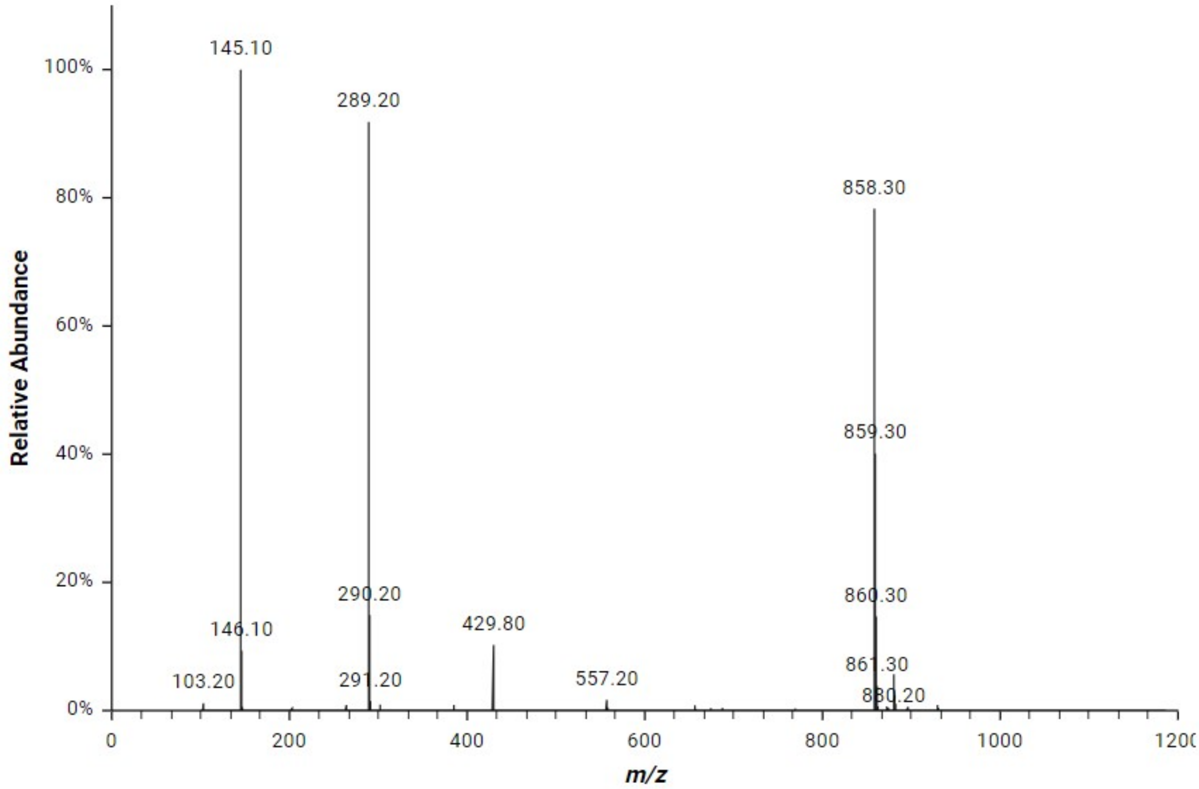

Supplementary Figure 25. Mass spectrum of crude β (34–42) peptide on DEG-PS using 2-MeTHF at 40 °C. Calculated for $C_{39}H_{71}N_9O_{10}S$: 858.11 Da, found: 858.30 $[M + H]^+$, 429.80 $[M + 2H]^{2+}$. Experimental conditions 1.

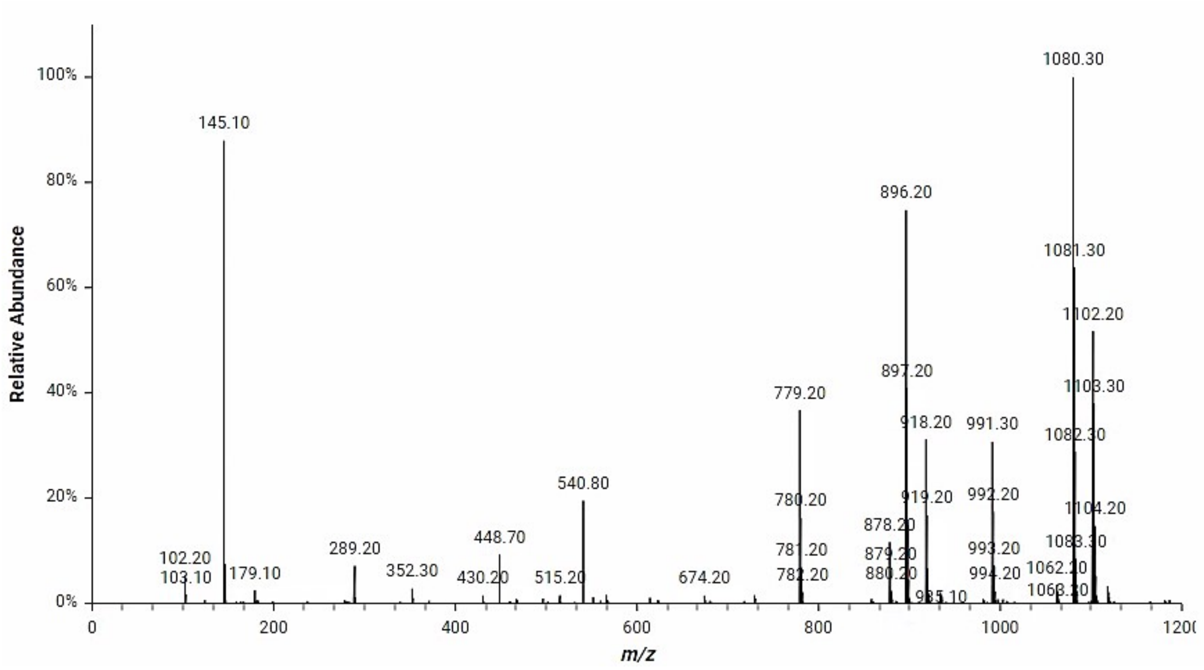

Supplementary Figure 26. Mass spectrum of crude Fmoc-β (34–42) peptide on DEG-PS using 2-MeTHF at 40 °C. Calculated for $C_{54}H_{81}N_9O_{12}S$: 1080.4 Da, found: 1081.3 $[M + H]^+$, 540.8 $[M + 2H]^{2+}$. Experimental conditions 1.

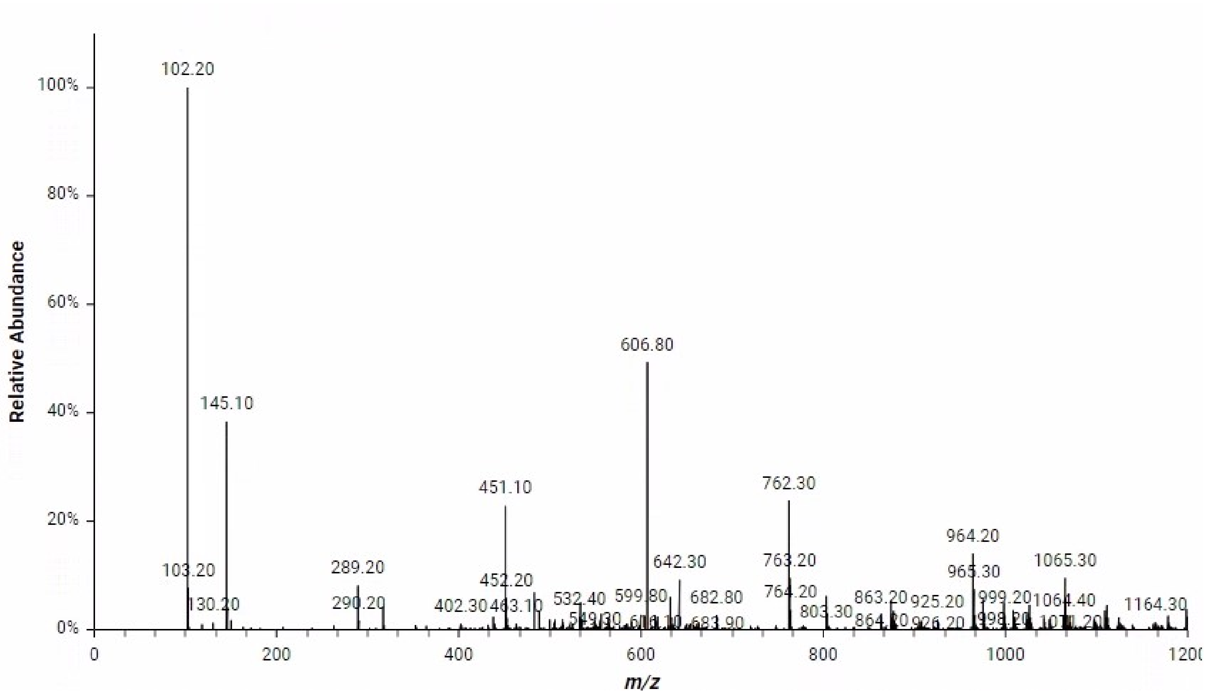

Supplementary Figure 27. Mass spectrum of crude JR peptide on DEG-PS using 2-MeTHF at 40 °C. Calculated for $C_{58}H_{89}N_{11}O_{15}S$: 1212.47 Da, found: 606.80 $[M + 2H]^{2+}$. Experimental conditions 1.

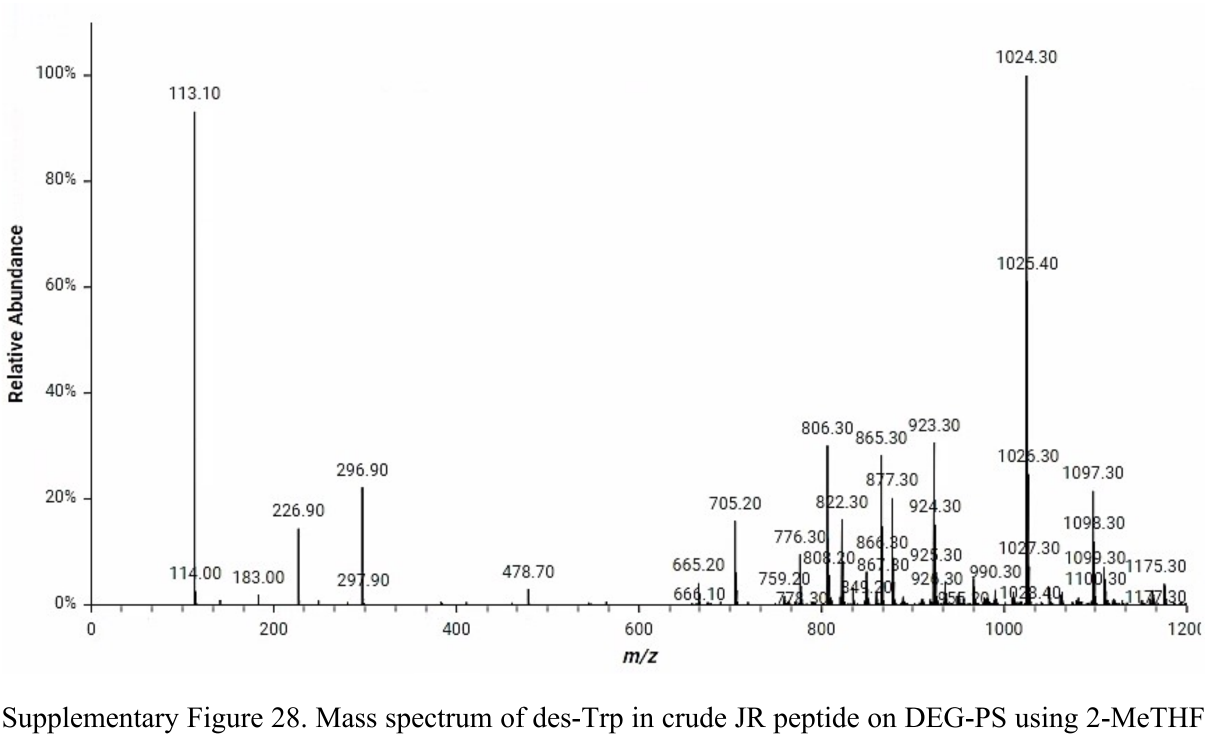

Supplementary Figure 28. Mass spectrum of des-Trp in crude JR peptide on DEG-PS using 2-MeTHF at 40 °C. Calculated for $C_{47}H_{79}N_9O_{14}S$: 1026.26 Da, found: 1027.30 $[M + H]^+$. Experimental conditions 1.

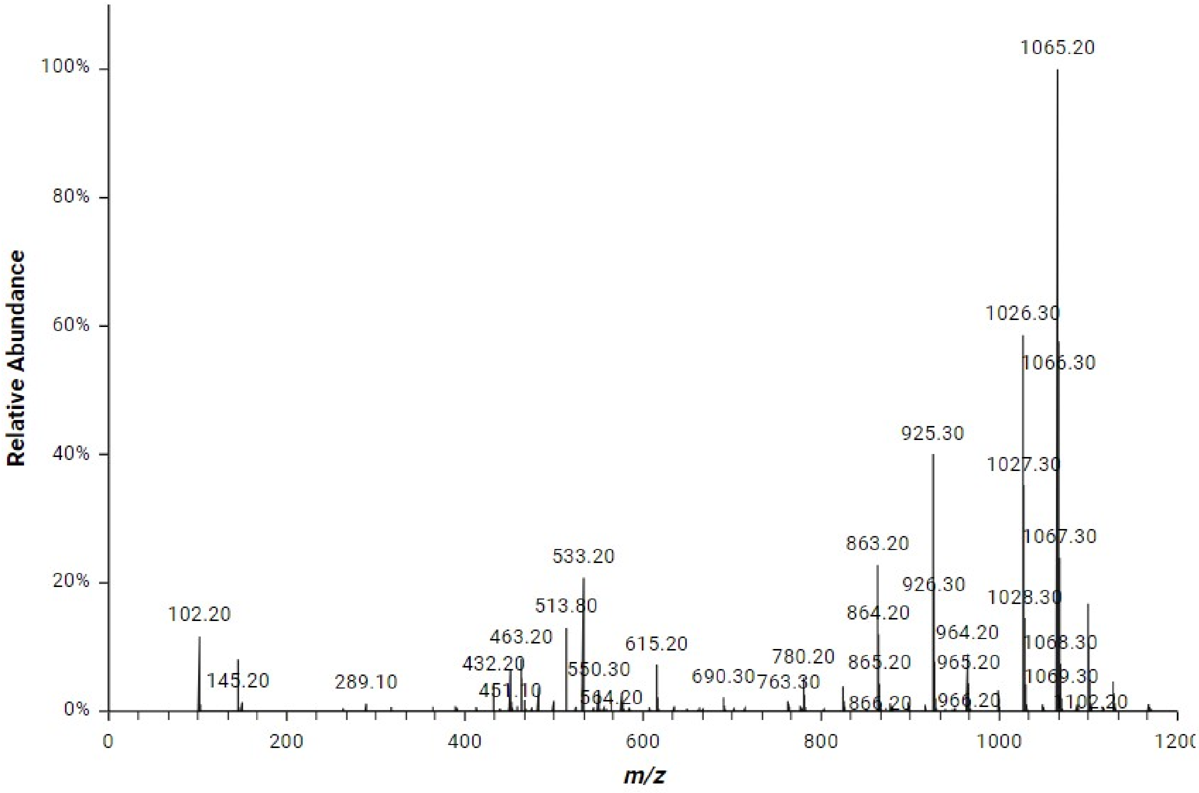

Supplementary Figure 29. Mass spectrum of des-Phe in crude JR peptide on DEG-PS using 2-MeTHF at 40 °C. Calculated for $C_{49}H_{80}N_{10}O_{14}S$: 1065.30 Da, found: 1065.30 $[M + H]^+$, 533.2 $[M + 2H]^{2+}$ Experimental conditions 1.

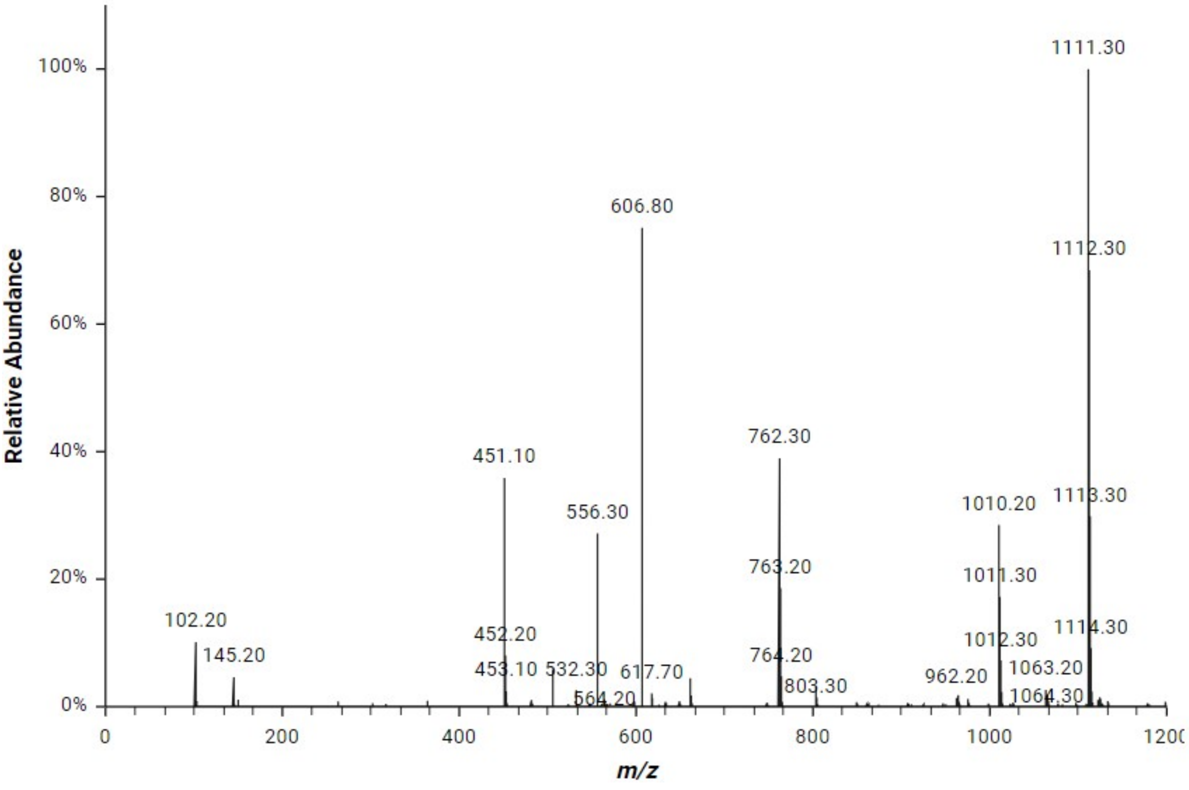

Supplementary Figure 30. Mass spectrum of des-Thr in crude JR peptide on DEG-PS using 2-MeTHF at 40 ^{o}C. Calculated for $C_{54}H_{82}N_{10}O_{13}S$: 1111.37 Da, found: 1112.30 $[M + H]^{+}$, 556.30 $[M + 2H]^{2+}$. Experimental conditions 1.

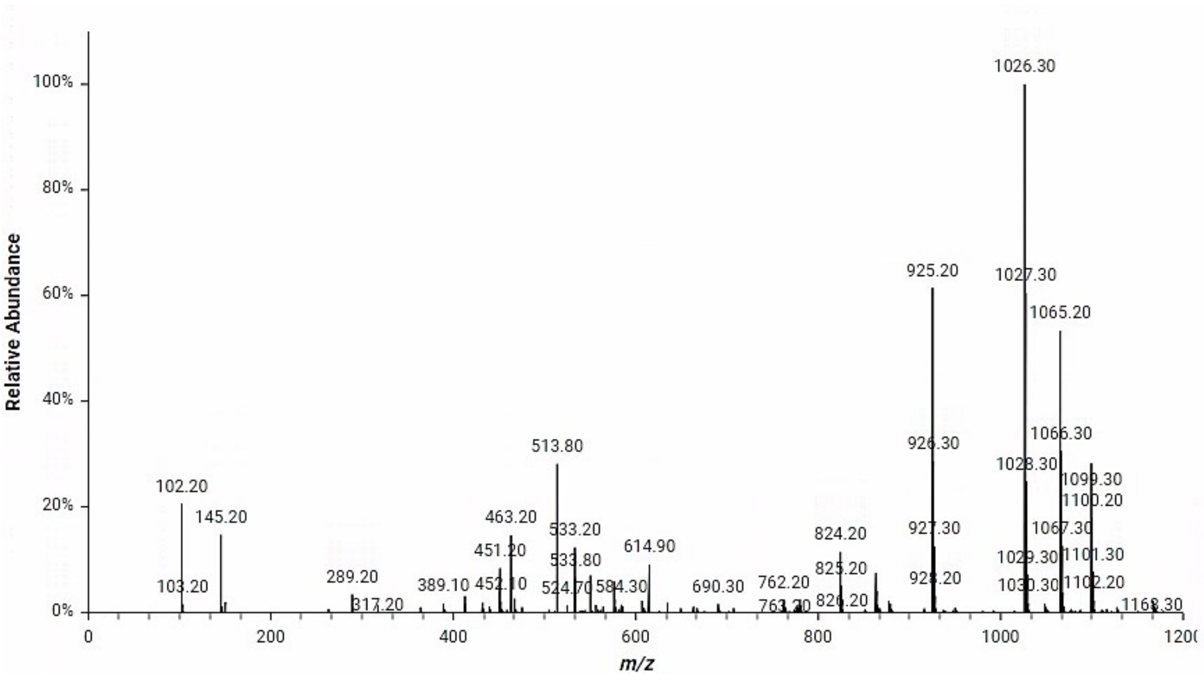

Supplementary Figure 31. Mass spectrum of des-Trp-Thr in crude JR peptide on DEG-PS using 2-MeTHF at 40°C. Calculated for $C_{43}H_{72}N_{8}O_{12}S$: 925.15 Da, found: 926.30 $[M + H]^{+}$. Experimental conditions 1.

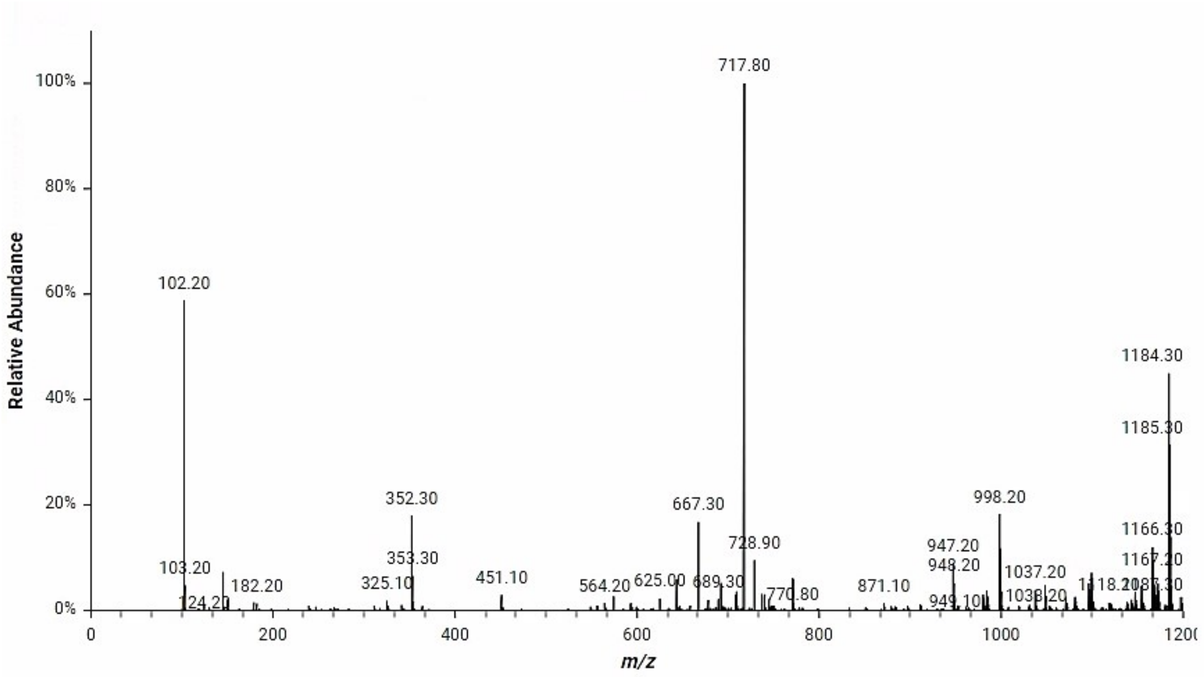

Supplementary Figure 32. Mass spectrum of crude Fmoc-JR peptide on DEG-PS using 2-MeTHF at 40°C. Calculated for $C_{73}H_{99}N_{11}O_{17}S$: 1434.72 Da, found: 717.80 $[M + 2H]^{2+}$. Experimental conditions 1.

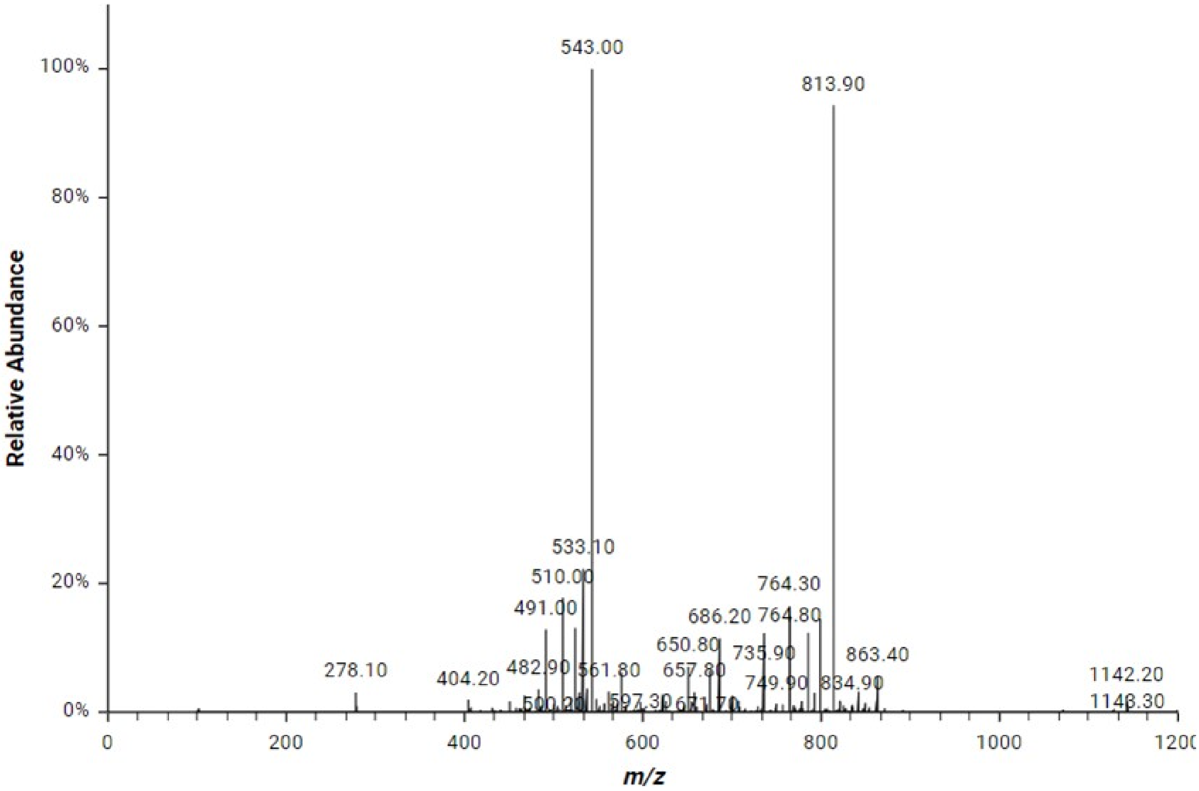

Supplementary Figure 33. Mass spectrum of crude ABRF1992 peptide on DEG-PS using 2-MeTHF at 40°C. Calculated for $C_{73}H_{106}N_{22}O_{21}$: 1627.78 Da, found: 813.90 $[M + 2H]^{2+}$, 543.00 $[M + 3H]^{3+}$. Des-Aib, calculated: 1006.1, found: 1007.3 $[M + H]^{+}$, 503.7 $[M + 2H]^{2+}$. Experimental conditions 1.

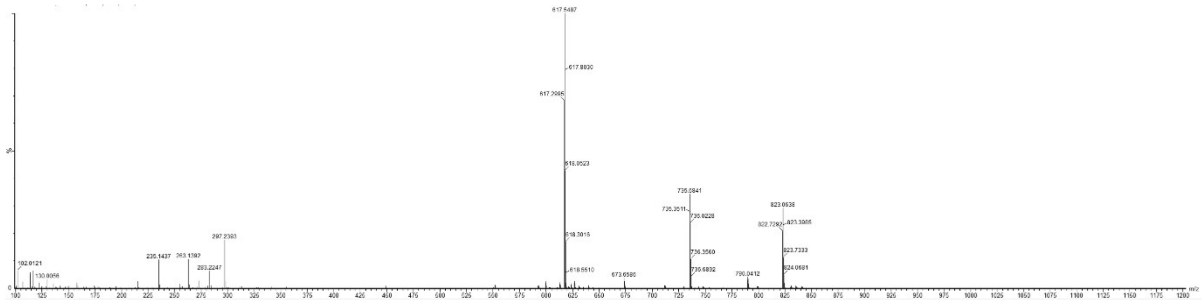

Supplementary Figure 34. High resolution mass spectrum of crude ABC analogue peptide on DEG-PS resin. $[M+3H]^{3+}$ calculated for $C_{110}H_{164}N_{30}O_{31}S_2$: 822.7285 Da, found: 822.7292 Da, Δ = 0.85 ppm, $[M+4H]^{4+}$ calculated: 617.2983 Da, found: 617.2995 Da Δ = 1.95 ppm. Experimental conditions 2.

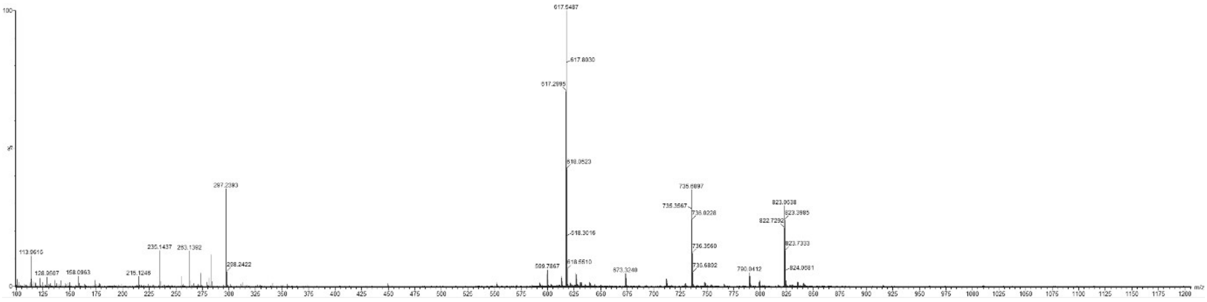

Supplementary Figure 35. High resolution mass spectrum of crude ABC analogue peptide on DVB-PS resin. $[M+3H]^{3+}$ calculated for $C_{110}H_{164}N_{30}O_{31}S2$: 822.7285 Da, found: 822.7292 Da, Δ = 0.85 ppm, $[M+4H]^{4+}$ calculated: 617.2983 Da, found: 617.2995 Da Δ = 1.95 ppm. Experimental conditions 2.

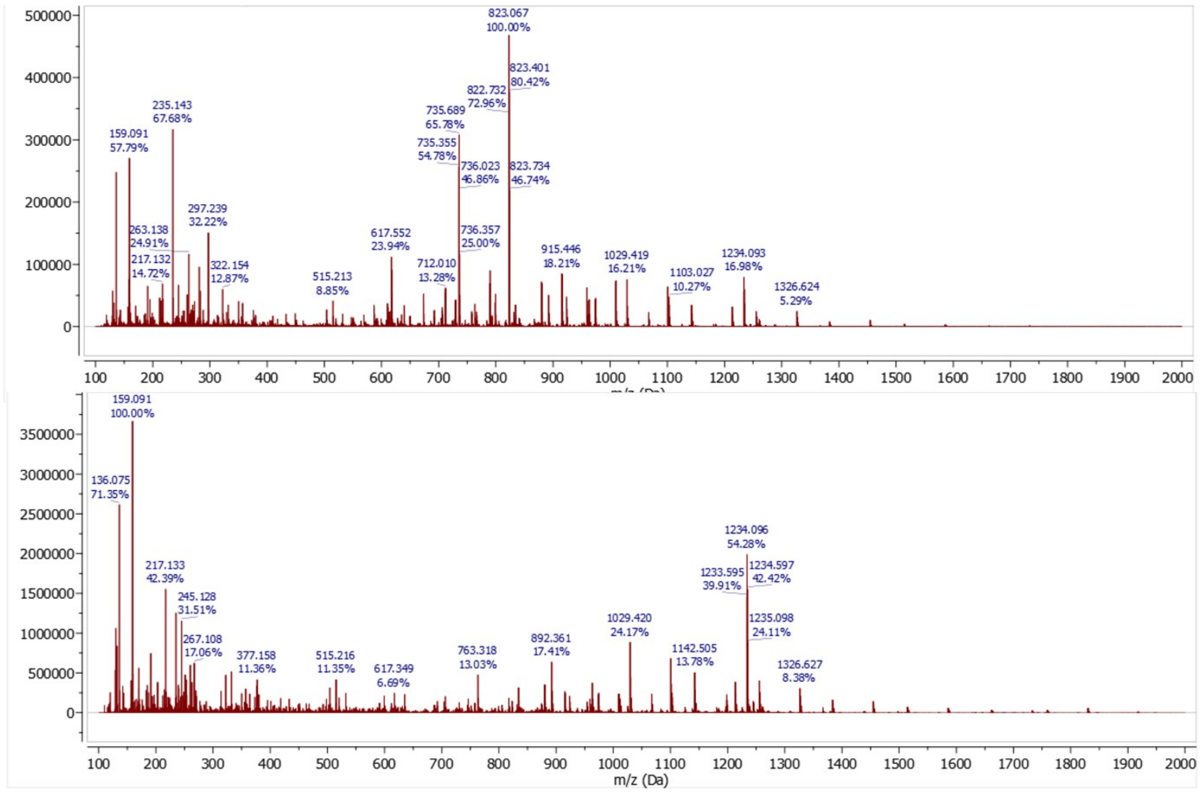

Supplementary Figure 36. MS^E spectra of ABC analogue peptide on DEG-PS resin, generated using conditions 2 with a collision voltage ramp of 15 V to 30 V (top) and 30 V to 60 V (bottom).

Supplementary Table 2: Fragment ions identified within the MS^E spectra obtained for the crude ABC analogue peptide.

Fragment ion	Calculated m/z	Observed m/z
B_2^+	263.139	263.138
B_3^+	449.218	449.218
Y_4^{++}	1009.483	1009.486
Y_5^+	1916.911	1916.927
Y_6^+	1829.879	1829.875
Y_7^+	1732.826	1732.845
Y_8^+	1585.757	1585.751
Y_9^+	1454.717	1454.722
Y_{10}^+	1326.622	1326.624
Y_{11}^+	1213.538	1213.539
Y_{12}^+	1100.454	1100.455
Y_{13}^+	963.395	963.397
Y_{14}^+	834.352	834.354
Y_{15}^+	706.294	706.296
Y_{17}^{++}	245.124	245.128

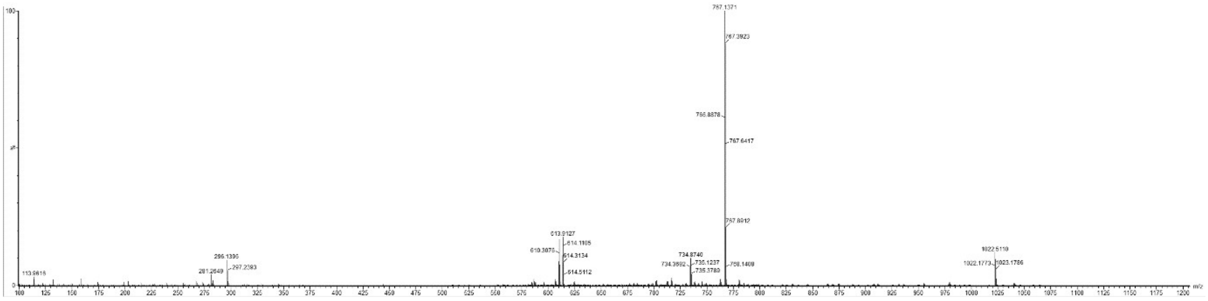

Supplementary Figure 37. High resolution mass spectrum of crude Thymosin peptide on DEG-PS resin. $[M+3H]^{3+}$ calculated for $C_{127}H_{214}N_{34}O_{53}$: 1022.1777 Da, found: 1022.1773 $[M + 3H]^{3+}$, Δ = 0.39 ppm. $[M+4H]^{4+}$ calculated: 766.8852 Da, found: 766.8878 $[M + 4H]^{4}+$ Δ = 3.39 ppm, $[M+5H]^{5+}$ calculated: 613.7097 Da, found: 613.7099, Δ = 0.33 ppm. Experimental conditions 2.

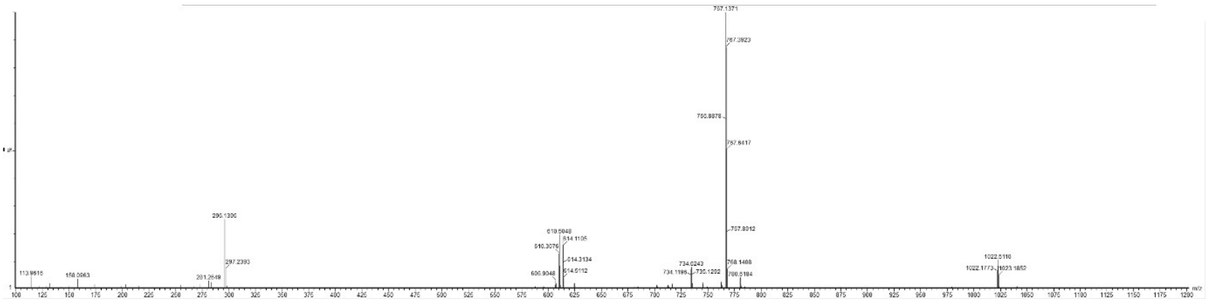

Supplementary Figure 38. High resolution mass spectrum of crude Thymosin peptide on DVB-PS resin. $[M+3H]^{3+}$ calculated for $C_{127}H_{214}N_{34}O_{53}$: 1022.1777 Da, found: 1022.1773 $[M + 3H]^{3+}$, Δ = 0.39 ppm. $[M+4H]^{4+}$ calculated: 766.8852 Da, found: 766.8878 $[M + 4H]^{4}+$ Δ = 3.39 ppm, $[M+5H]^{5+}$ calculated: 613.7097 Da, found: 613.7099, Δ = 0.33 ppm. Experimental conditions 2.

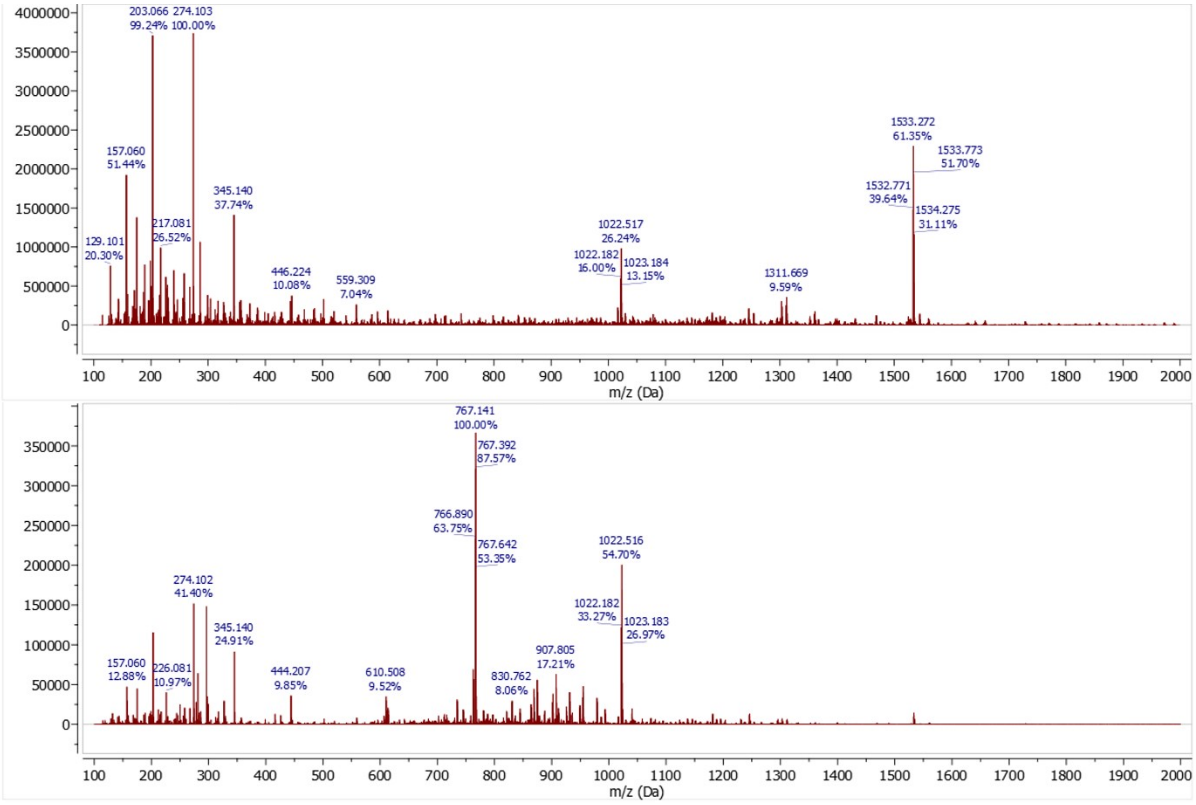

Supplementary Figure 39. High resolution MS^E spectra of crude Thymosin peptide on DEG-PS resin, generated using conditions 2 with a collision voltage ramp of 15 V to 30 V (top) and 30 V to 60 V (bottom).

Supplementary Table 3: Fragment ions identified within the MS^E spectra obtained for the crude Thymosin peptide.

Fragment ion	Calculated m/z	Observed m/z
B_2^+	203.066	203.066
B_3^+	274.103	274.103
B_4^+	345.141	345.140
B_5^+	444.209	444.208
B_6^+	559.236	559.237
Y_7^{++}	1254.140	1254.144
Y_{15}^{++}	830.425	830.433
B_{24}^+	1302.666	1302.659
